# Comparative cardiomyocyte differentiation potential of rat adipose-derived mesenchymal stem cells from two anatomical sites: metabolomic profiling and pathway analysis

**DOI:** 10.3389/fcell.2025.1604605

**Published:** 2025-06-19

**Authors:** Ahmed Farag, Haney Samir, Sai Koung Ngeun, Masahiro Kaneda, Hanan Hendawy, Ken Takahashi, Ryou Tanaka

**Affiliations:** ^1^ Faculty of Agriculture, Veterinary Teaching Hospital, Tokyo University of Agriculture and Technology, Tokyo, Japan; ^2^ Department of Surgery, Anesthesiology, and Radiology, Faculty of Veterinary Medicine, Zagazig University, Zagazig, Egypt; ^3^ Department of Theriogenology, Faculty of Veterinary Medicine, Cairo University, Giza, Egypt; ^4^ Department of Neurophysiology, National Center of Neurology and Psychiatry, Tokyo, Japan; ^5^ Laboratory of Veterinary Anatomy, Division of Animal Life Science, Tokyo University of Agriculture and Technology, Tokyo, Japan; ^6^ Department of Veterinary Surgery, Faculty of Veterinary Medicine, Suez Canal University, Ismailia, Egypt; ^7^ Department of Pediatrics, Juntendo University, Urayasu Hospital, Chiba, Japan

**Keywords:** adipose-derived mesenchymal stem cells, cardiomyocyte differentiation, 5-azacytidine, metabolomic profiling, regenerative medicine

## Abstract

**Background:**

Adipose-derived mesenchymal stem cells (AD-MSCs) have emerged as a promising source for cardiac regenerative therapy due to their multipotency and ease of isolation. However, the impact of anatomical origin on their cardiomyocyte differentiation potential remains unclear. Metabolic analysis provides valuable real-time insights into the cellular metabolic state, capturing dynamic changes in metabolite concentrations that reflect both internal cellular mechanisms and external stimuli. This approach allows us to identify specific metabolic pathways activated during cardiomyocyte differentiation, offering a deeper understanding of how the anatomical origin of stem cells influences their differentiation potential and metabolic flexibility. Such insights are critical for optimizing stem cell-based therapies for cardiac regeneration. This study aimed to compare the differentiation capacity of AD-MSCs derived from peri-ovarian and peri-renal adipose tissue, with a focus on metabolic adaptations during cardiomyocyte differentiation.

**Methods:**

AD-MSCs were isolated from peri-ovarian and peri-renal fat of Sprague-Dawley rats and characterized by morphology, immunophenotyping, and multilineage differentiation potential. Cardiomyocyte differentiation was induced using 5-azacytidine, and morphological changes were assessed via phase-contrast microscopy and immunofluorescence staining for cardiac troponin T (cTnT). Untargeted metabolomic profiling was performed using gas chromatography-mass spectrometry (GC-MS), followed by principal component analysis (PCA), partial least squares discriminant analysis (PLS-DA), and pathway enrichment analysis.

**Results:**

Both peri-ovarian and peri-renal AD-MSCs exhibited similar fibroblast-like morphology, MSC-specific marker expression (CD44, CD90, CD29), and multilineage differentiation potential. Following cardiomyocyte induction, both groups displayed morphological changes indicative of differentiation and strong cTnT expression. Metabolomic analysis of the cardiogenic differentiation samples identified distinct metabolic adaptations between the two AD-MSC sources. Peri-ovarian AD-MSCs exhibited a broader metabolic reprogramming, with increased engagement of glycolysis, fructose metabolism, glycerolipid metabolism, and the TCA cycle, suggesting enhanced metabolic flexibility and energy efficiency. In contrast, peri-renal AD-MSCs relied more on galactose metabolism, indicating an alternative energy strategy during differentiation.

**Conclusion:**

The anatomical origin of AD-MSCs influences their metabolic landscape during cardiomyocyte differentiation. Peri-ovarian AD-MSCs demonstrated greater metabolic adaptability, potentially favoring their differentiation capacity, making them a promising candidate for cardiac regenerative applications.

## 1 Introduction

Stem cells represent a vital reservoir of undifferentiated, self-renewing cells with the remarkable ability to differentiate into diverse specialized cell lineages. This unique multipotency positions them as a promising tool for regenerative medicine, enabling the repair and regeneration of damaged tissues. The differentiation potential of stem cells is well-established; however, the efficiency of this process can be influenced by various factors, including the specific stem cell type and the protocols employed (Burridge et al., 2012; Hartman et al., 2016; Farag et al., 2025). Among the various types of stem cells, mesenchymal stem cells (MSCs) have gained significant attention due to their ability to differentiate into tissues from both mesodermal and non-mesodermal origins. Additionally, their unique immune-modulatory properties make them especially suitable for allogeneic transplantation, positioning them as an attractive option for “off-the-shelf” therapies (Ankrum et al., 2014; Omar et al., 2019).

MSCs are easily obtainable from multiple tissue types, such as bone marrow, adipose tissue, peripheral blood, and various other adult somatic tissues, each offering distinct growth characteristics and differentiation potentials (Bianco, 2014; Farag et al., 2025). Recent studies, however, have increasingly emphasized adipose tissue as a particularly valuable source for obtaining multipotent cells, specifically adipose-derived stem cells (AD-MSCs) (Lee et al., 2004). Multiple factors drive this preference, chief among them being the abundance, accessibility, and expandable nature of adipose tissue as a rich reservoir of mesenchymal stem cells. Secondly, standard adipose tissue extraction techniques facilitate the efficient isolation of a large number of cells. Thirdly, AD-MSCs demonstrate superior *in vitro* proliferation rates when compared to bone marrow-derived stem cells. Furthermore, these cells have shown potential for differentiating into endothelial and cardiomyocyte phenotypes in small animal models, as highlighted by several studies (Planat-Benard et al., 2004b; 2004a; Gimble et al., 2007; Shi et al., 2012; Vishnubalaji et al., 2012).

Cardiomyocyte differentiation primarily occurs during the perinatal stages of life. However, in adulthood, the regenerative capacity of cardiac tissue is limited, which proves inadequate for compensating for degeneration in conditions such as myocardial infarction (Pfeffer and Braunwald, 1990). As a potential solution to this limitation, cell transplantation has been explored as a therapeutic alternative (Orlic et al., 2002). This has prompted extensive research focused on identifying suitable sources for transplanted cells and understanding the mechanisms behind cardiomyocyte proliferation and differentiation. Various sources, including embryonic, fetal, and adult cells, have been evaluated through experimentation (Makino et al., 1999; Condorelli et al., 2001; Jackson et al., 2001; Orlic et al., 2001; Grounds et al., 2002; Planat-Benard et al., 2004a). Mesenchymal stem cells (MSCs) derived from adipose tissue have garnered attention for their ability to differentiate into functional cardiomyocytes. Successful differentiation of AD-MSCs into cardiomyocytes has been demonstrated both *in vitro* and *in vivo* (Bai and Alt, 2010; Kakkar et al., 2019; Hasani et al., 2020; Farag et al., 2024b). This positions adipose tissue-derived MSCs as promising candidates to address the challenges posed by limited cardiac tissue regeneration in pathological conditions.

Metabolomics has emerged as a powerful tool in systems biology, offering novel insights into the functional dynamics of stem cells (SCs). While genomics and proteomics have long been employed in SC research, metabolomics provides a distinct perspective by capturing real-time metabolic activity, enabling a deeper understanding of cellular behavior and regulatory mechanisms (Martano et al., 2019). This is because metabolomics not only reflects upstream cellular features (such as genes, RNA, and proteins) but also serves as an effective probe for external stimuli and the interactions between stem cells and their surrounding microenvironment (Bispo et al., 2021).

Metabolomics encompasses the systematic identification and quantification of low-molecular-weight endogenous compounds (metabolites) present in biological specimens. This analytical approach primarily employs high-throughput platforms like Nuclear Magnetic Resonance (NMR) spectroscopy and Mass Spectrometry (MS), often coupled with multivariate statistical analysis (MVA) for data interpretation and pattern recognition (Segers et al., 2019). As a global tool for analyzing the cellular metabolic state, metabolomics has proven valuable in monitoring cell cultures (Vernardis et al., 2013) and studying stem cell metabolic physiology (McNamara et al., 2012), thereby shedding light on the underlying metabolic networks. One of the strengths of metabolomics lies in its ability to conduct multivariate analysis, which captures correlations between metabolic pathways that are often overlooked by traditional enzyme activity assays targeting only a limited number of enzymes or specific metabolites. The sensitivity of metabolomics allows for the detection of minute changes in metabolite concentrations, which could otherwise be missed in one-at-a-time analyses (Klontzas et al., 2017).

However, there is currently no study focused on identifying alterations in the metabolite profiles of stem cells during cardiomyocyte differentiation using the GC-MS technique, previous research has explored the relationship between various harvesting sites of visceral fat and the morphology, immunophenotype, pluripotency, and multilineage differentiation potential of adipose-derived mesenchymal stem cells (AD-MSCs) (Kaewkhaw et al., 2011; Megaloikonomos et al., 2018; Hendawy et al., 2021). The key fact that needs to be understood is how the anatomical origin of AD-MSCs influences their cardiomyocyte differentiation potential. This includes identifying the metabolic pathways that are activated or altered during differentiation, as well as any differences in the efficiency or mechanism of differentiation between AD-MSCs harvested from different anatomical sites.

Adipose tissue depots exhibit anatomical and functional heterogeneity, which may influence the differentiation potential of resident mesenchymal stem cells (Zuk et al., 2002; Pachón-Peña et al., 2011). Peri-ovarian and peri-renal adipose tissues were selected for this study due to their distinct embryological origins, vascularization patterns, and metabolic roles. Peri-ovarian adipose tissue, associated with reproductive hormonal regulation, demonstrates high metabolic plasticity, while peri-renal adipose tissue, encasing the kidneys, is implicated in systemic lipid metabolism and hypertension (Fuente-Martín et al., 2013; Hendawy et al., 2021). Prior studies suggest that anatomical location impacts MSC properties, including proliferation rates and lineage-specific differentiation (Baer and Geiger, 2012; Megaloikonomos et al., 2018). We hypothesized that these functional and microenvironmental differences might translate to divergent metabolic adaptations during cardiomyocyte differentiation. From the metabolomic analysis, we aim to identify the specific metabolic pathways that are activated or altered during cardiomyocyte differentiation of AD-MSCs from different anatomical sites. This includes understanding the key metabolites that are upregulated or downregulated during differentiation, as well as how these metabolic changes influence the differentiation process. Additionally, we seek to clarify whether there are significant differences in the metabolic profiles of AD-MSCs from peri-ovarian and peri-renal adipose tissue, and how these differences might contribute to variations in their differentiation potential. To achieve this, we employed comprehensive metabolomic profiling to evaluate the metabolic shifts that occur during the differentiation process, offering a novel perspective on the cardiomyocyte differentiation potential of AD-MSCs from different anatomical sites.

## 2 Materials and methods

### 2.1 Animal model and housing conditions

We utilized five healthy, pathogen-free female Sprague-Dawley rats, each ranging in age from six to 8 weeks and weighing between 250 and 300 g. The deliberate selection of female rats was made to facilitate the acquisition of both peri-ovarian and peri-renal adipose tissue. These rats were housed in cages within a carefully regulated environment, maintaining a temperature range of 20°C–25°C, humidity levels between 40% and 60%, and adhering to a 12-h light/dark cycle. The rats were provided with a consistent supply of dry food and water. Euthanasia was performed using the inhalation method with an overdose of isoflurane, following the protocol described by Siennicka et al. (2021). Additionally, the animal study was approved by the Animal Care and Use Committee of Tokyo University of Agriculture and Technology. All procedures adhered to the institutional guidelines (Approval Numbers: R05-158 and R03-163).

### 2.2 Isolation and processing of adipose-derived mesenchymal stem cells

Adipose tissue was aseptically harvested from two visceral depots in rats (peri-ovarian and peri-renal regions). The collected specimens were processed under sterile conditions through the following protocol: First, tissues were washed extensively with phosphate-buffered saline (PBS; Medicago AB, Uppsala, Sweden, cat. no. 09-8912-100) and mechanically dissociated using sterile scissors in a laminar flow hood. The minced tissue fragments were then digested in Hank’s balanced salt solution (HBSS; Thermo Fisher Scientific, cat. no. 14025-092) supplemented with 0.1% collagenase type I (Gibco, cat. no. 17100017) for 60 min at 37°C with constant agitation.

The enzymatic reaction was terminated by adding chilled HBSS, followed by centrifugation (800 × g, 10 min) to obtain a stromal vascular fraction pellet. Cellular aggregates were removed by filtration through a 100 μm mesh (BD Falcon), and erythrocyte contamination was addressed using 1 mL of specialized lysis buffer (eBioscience™ Promega, cat. no. 00-4333–57). The final cell pellet was reconstituted in complete culture medium consisting of DMEM (FUJIFILM Wako, cat. no. 043-30085) supplemented with 10% FBS (COSMOBIO, cat. no. CCP-FBS-BR-500), 1% non-essential amino acids (FUJIFILM Wako, cat. no. 139-15651), and 1% penicillin-streptomycin (FUJIFILM Wako, cat. no. 161-23181) (Fathi and Farahzadi, 2017).

### 2.3 *In vitro* culturing and maintenance of rat AD-MSCs

Isolated cells were plated in 100-mm culture dishes (Nippon Genetics, cat. no. TR4002) and maintained under standard culture conditions (37°C, 5% CO_2_, 95% humidity). The culture medium was refreshed every 72 h to maintain optimal growth conditions until cells reached 80% confluence. For subculturing, adherent cells were detached using 0.25% trypsin (37°C incubation) and neutralized with complete DMEM containing 10% FBS. Cells were then reseeded at appropriate densities for subsequent passages. All experiments were conducted using third-passage (P3) cells to ensure consistency in cellular responses.

### 2.4 Characterization of cultured AD-MSCs

#### 2.4.1 Immunophenotyping

Cell morphology was assessed daily using an inverted phase-contrast microscope (Olympus CKX31, Tokyo, Japan) to monitor characteristic mesenchymal stem cell (MSC) features. For immunophenotypic characterization, cells were analyzed via flow cytometry using established MSC markers (CD44, CD90, CD29) along with the hematopoietic lineage marker CD45 to confirm MSC purity and exclude hematopoietic contamination (Kaewkhaw et al., 2011; Hendawy et al., 2021; Farag et al., 2024a). Third-passage (P3) cells were enzymatically detached using trypsin and stained with PE-conjugated antibodies according to manufacturer-recommended dilutions (1:100): CD44 (Abcam, ab23396), CD90 (BD Biosciences, 551401), CD29 (BD Biosciences, 562154), and CD45 (BioLegend, 202207). Following a 30-min incubation at 4°C in the dark, cells were washed with PBS and analyzed using a CytoFLEX flow cytometer (Beckman Coulter). Isotype-matched antibodies served as negative controls for gating strategy implementation.

#### 2.4.2 Tri-lineage differentiation potential of AD-MSCs

The multi-differentiation potential of AD-MSCs harvested from both sites was assessed by inducing the cells to differentiate into osteogenic, chondrogenic, and adipogenic lineages, as previously described (Lotfy et al., 2014; Hendawy et al., 2021).

##### 2.4.2.1 Osteogenic differentiation

Third-passage AD-MSCs were trypsinized and plated in 6-well plates at a density of 1 × 10^5^ cells/well. At 80% confluence, cells were divided into two groups: (1) experimental wells (n = 4) receiving osteogenic induction medium (DMEM with 10% FBS, 100 nM dexamethasone [Sigma-Aldrich D4902], 0.2 mM ascorbic acid [Wako 016-04805], and 10 mM β-glycerophosphate [Sigma-Aldrich G9422-10G]), and (2) control wells (n = 2) maintained in standard growth medium. Media were replaced twice weekly for 21 days. Osteogenic differentiation was confirmed by calcium deposition detection using Alizarin Red S staining (Sigma-Aldrich 40-1009-5), with positive differentiation indicated by distinct extracellular matrix mineralization (red staining).

##### 2.4.2.2 Chondrogenic differentiation

Third-passage AD-MSCs were plated at a density of 1 × 10^5^ cells/well in 6-well plates and maintained in serum-free chondrogenic induction medium (PromoCell C-28012, Heidelberg, Germany). The induction medium was replaced every 48-72 h throughout the 21-day differentiation period. Chondrogenic differentiation was assessed through Alcian Blue 8GX staining (Sigma-Aldrich 66011), which specifically detects sulfated proteoglycans characteristic of cartilage extracellular matrix. Positive chondrogenesis was confirmed by intense blue staining indicative of proteoglycan accumulation.

##### 2.4.2.3 Adipogenic differentiation

Third-passage AD-MSCs were plated at a density of 1 × 10^5^ cells/well in 6-well plates. At 80% confluence, cells were divided into: (1) Induction group (n = 4 wells): Adipogenic differentiation medium (DMEM with 10% FBS, 1 μM dexamethasone, 500 μM IBMX [Sigma I5879], 100 μM indomethacin [Sigma I7378], and 5 μg/mL insulin [Sigma I5500]) (2) Control group (n = 2 wells): Standard growth medium.

Medium was replaced twice weekly for 14 days. Adipogenic differentiation was assessed by Oil Red O staining (Sigma O0625) of intracellular lipid vacuoles, with positive differentiation indicated by distinct, red-stained lipid droplets under microscopic examination.

### 2.5 Induction of cardiomyocyte differentiation in AD-MSCs

AD-MSCs from two distinct sites (peri-ovarian and peri-renal) at passage three were cultured in 6-well plates until they reached 80% confluency. To induce their differentiation into cardiomyocytes, the AD-MSCs were resuspended in a cell medium supplemented with 10 μmol/L of 5-Azacytidine (cat. no. A2385, Sigma-Aldrich, Tokyo, Japan). Following a 24-h incubation period, the cells were washed twice with PBS, and the medium was replaced with a complete medium devoid of 5-Azacytidine (Antonitsis et al., 2007; Xing et al., 2012a). Cardiomyocyte differentiation was induced using 10 μmol/L 5-azacytidine, a concentration selected based on extensive optimization and prior studies demonstrating its efficacy in promoting cardiomyogenic differentiation of MSCs without excessive cytotoxicity (Antonitsis et al., 2007; Xing et al., 2012a; Eldin et al., 2019; Farag et al., 2024a). Lower concentrations have shown inconsistent differentiation efficiency (Makino et al., 1999). While the effects of 5-azacytidine are dose-dependent, this intermediate concentration has consistently been shown to promote cardiac marker expression, including cTnT, while maintaining acceptable cell viability. Further investigation into dose-response effects and cytotoxic thresholds is warranted for future optimization.

#### 2.5.1 Morphological analysis of cardiomyocyte differentiation

The analysis of cell morphology and shapes at different time points post-induction was conducted on the cultured cells using an inverted microscope (Makino et al., 1999; Xing et al., 2012b).

#### 2.5.2 Immunofluorescence staining

Following a 21-day differentiation period, immunofluorescence analysis was performed to evaluate cardiomyocyte differentiation in AD-MSCs derived from both anatomical locations by detecting cardiac troponin T (cTnT), a specific cardiac marker (Yang et al., 2012). Cell fixation was performed using 4% paraformaldehyde (NACALAI TESQUE, 09154-85) for 15 min at 4°C. Cells were then permeabilized with 0.5% Triton X-100 (Sigma-Aldrich, 9036-19–5) for 10 min, followed by blocking with 1% BSA (Sigma-Aldrich, A9418-5G) for 1 h at room temperature. The samples were incubated overnight at 4°C with anti-cTnT primary antibody (Abcam ab209813) diluted 1:400 in blocking solution (Yi et al., 2017). Following primary antibody incubation, cells were treated with FITC-labeled secondary antibody (Abcam ab6717, 1:1000 dilution) for 60 min at ambient temperature, with light protection implemented throughout the process. Nuclear counterstaining was subsequently performed using DAPI solution (Invitrogen D3571, 5 μg/mL concentration, 1:1000 dilution) for a 5-min duration (Hao et al., 2018). The resultant plates were captured using a fluorescence microscope BZ-9000 (KEYENCE Instruments Inc., Tokyo, Japan).

#### 2.5.3 Metabolomic assessment of cell cultures

Cell culture specimens were subjected to untargeted metabolomic profiling employing gas chromatography-mass spectrometry (GC/MS) according to established protocols (Xia and Wishart, 2011; Pu et al., 2019; Kusama et al., 2022; Samir et al., 2023; 2025) with slight modifications. Briefly, 994 μL of extraction solvent (methanol: pure water: chloroform = 2.5:1:1) was added to a tube containing approximately 10 mg of isolated cells and 6 μL of 2-isopropylmalic acid (1 mg/mL; Sigma-Aldrich, St. Louis, MO, United States). The mixture was vortexed and thoroughly homogenized. The tubes were incubated in a thermo-shaker at 37°C and 1200 rpm for 30 min, followed by centrifugation at 4°C and 16,000 g for 5 min.

Next, 900 μL of the supernatant was transferred to another 2 mL tube, and 450 μL of chloroform was added. The tubes were again centrifuged at 4°C and 16,000 g for 5 min. Approximately 500 μL of the upper layer was transferred to a new 1.5 mL tube. After adding 200 μL of pure water, the tubes were centrifuged again under the same conditions, and 500 μL of the upper layer was collected into a new 1.5 mL tube to prepare the quality control (QC) set.

All sample tubes, including quality control (QC) aliquots, were concentrated using a Yamato RD-400 centrifugal evaporator (60 min) followed by overnight lyophilization. The dried residues were derivatized with 80 μL of 20 mg/mL methoxyamine-pyridine solution (Sigma-Aldrich), vortex-mixed, and incubated at 37°C (1200 rpm, 90 min) in a thermo-shaker. Following this, 40 μL of N-methyl-N-trimethylsilyl-trifluoroacetamide (GL Science) was added, with additional incubation under identical conditions (30 min). After centrifugation (16,000 × g, 5 min, 20°C), the clarified supernatant was transferred to GC/MS vials for analysis (Shimadzu system).

To ensure the reliability of our metabolomic results, we analyzed 5 biological replicates per group (derived from individual rats), with each sample measured in triplicate to assess technical precision. We included pooled QC samples every fifth run to monitor instrument stability, excluding metabolites with >30% variability. Data were normalized using internal standards, processed in MetaboAnalyst 6.0, and filtered for missing values. These steps align with standard metabolomics practices to minimize batch effects and ensure reproducibility (Dunn et al., 2011).

### 2.6 Statistical analysis

A Student’s t-test was used to analyze MSC-specific cell surface markers, while a one-way ANOVA was applied to assess variations between the two AD-MSC harvesting sites. All statistical analyses were conducted using GraphPad Prism 8.0 (GraphPad Software, San Diego, CA, United States). A p-value of <0.05 was considered statistically significant, and a p-value of >0.05 was regarded as non-significant.

Metabolomic profiling was performed across four experimental groups (peri-ovarian control, peri-ovarian induced, peri-renal control, and peri-renal induced) using MetaboAnalyst 6.0. Raw peak intensity data were normalized against 2-isopropyl malic acid and processed with default platform settings. Univariate analysis employed ANOVA (p < 0.05) with Fisher’s LSD *post hoc* testing, while multivariate approaches included PCA (top five score plots in 2-D and biplot formats) and PLS-DA (top 25 metabolites per group). Hierarchical clustering heatmaps (samples × features) visualized concentration patterns, followed by pathway enrichment analysis using SMPDB libraries and chemical structure-based classification. All analyses adhered to established protocols (Xia and Wishart, 2011; Kusama et al., 2022; Samir et al., 2023) within the MetaboAnalyst environment.

## 3 Results

### 3.1 Morphological characterization of AD-MSCs

**FIGURE 1:**
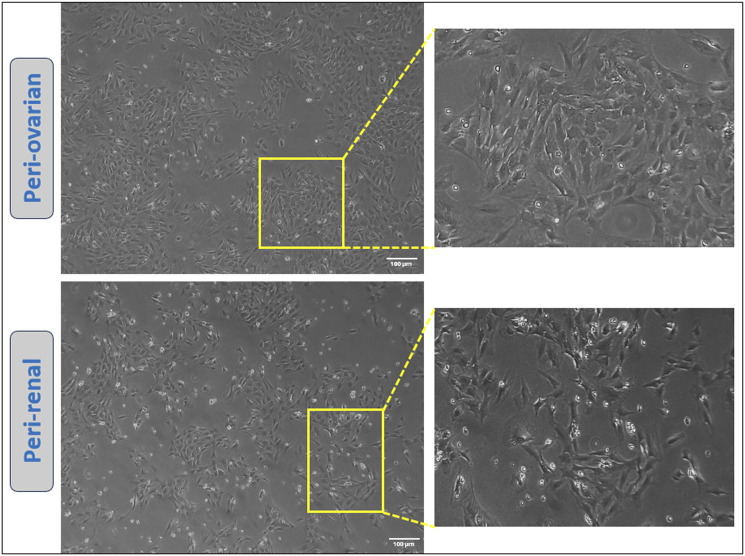
Representative images showcasing the morphology of cultured AD-MSCs obtained from two distinct adipose tissue depots at passage three. Scale bar: 100 μm.

Adipose-derived mesenchymal stem cells (AD-MSCs) isolated from different anatomical sites demonstrated a characteristic adherence to plastic culture plates, readily observed under an inverted microscope. The majority of the cells exhibited a fibroblast-like morphology, which is typical of mesenchymal stem cells. Notably, AD-MSCs from both peri-ovarian and peri-renal adipose tissues displayed consistent fibroblastic morphology, with no discernible differences between the two groups, as illustrated in Figure 1.

### 3.2 Immunophenotypic profile of AD-MSCs at passage 3

**FIGURE 2:**
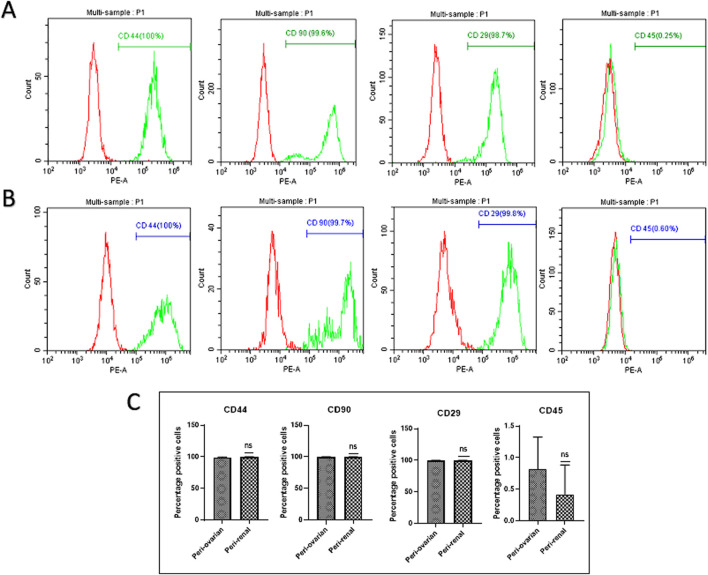
Comparison of Cell Surface Marker Expression in Third-Passage AD-MSC Cultures from Two Anatomical Sites. Representative histograms depict the expression of surface markers in AD-MSCs harvested from peri-ovarian and peri-renal adipose tissues. These cells demonstrated positive expression for mesenchymal stem cell-specific markers CD44, CD90, and CD29, while lacking expression of the hematopoietic marker CD45. **(A)** Peri-Ovarian site. **(B)** Peri-Renal site. **(C)** Combined analysis. Values are presented as mean ± SD, with no significant differences observed in surface marker expression between the two anatomical sites.

AD-MSCs from both harvest sites exhibited positive expressions for mesenchymal markers CD44, CD90, and CD29, with no significant differences between the two anatomical locations. Conversely, these cells consistently lacked expression of the hematopoietic marker CD45, with no variations observed between the two groups. (Figure 2).

### 3.3 Multilineage differentiation potential of AD-MSCs

The multilineage differentiation potential of AD-MSCs from peri-ovarian and peri-renal adipose tissues was evaluated under specific *in vitro* conditions, demonstrating their ability to differentiate into osteocytes, chondrocytes, and adipocytes. Osteogenic differentiation was evident after 21 days of culture in osteogenic medium, as cells exhibited elongated, spindle-shaped morphology and progressively formed calcified extracellular matrix deposits. Alizarin Red S (ALZ) staining confirmed increased matrix mineralization, a hallmark of osteogenic commitment.

**FIGURE 3:**
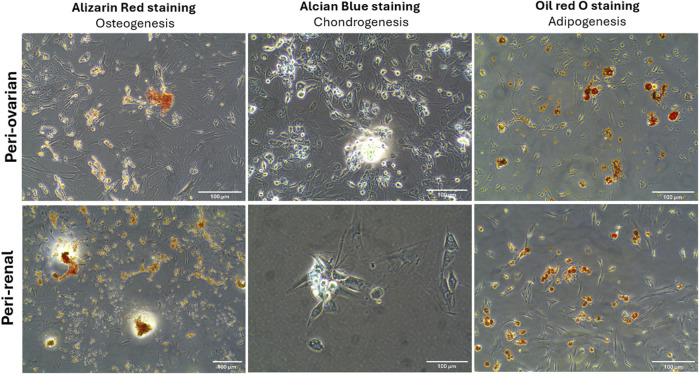
Multilineage differentiation potential of AD-MSCs from peri-ovarian and peri-renal adipose tissue. Light microscopy images demonstrate the differentiation of third-passage AD-MSCs into osteogenic, chondrogenic, and adipogenic lineages under specific induction conditions. Scale bar: 100 μm.

Chondrogenic differentiation was successfully induced using a complete chondrogenic medium containing established chondrogenic agents. Positive Alcian Blue staining revealed the presence of cartilaginous matrix components, demonstrating the ability of AD-MSCs to commit to the chondrogenic lineage. Morphologically, the cells became round with low cell density and extensive deposition of a cartilaginous matrix. Similarly, adipogenic differentiation was observed when AD-MSCs were cultured in adipogenic medium, as indicated by Oil Red O staining. The staining demonstrated the formation of intracellular lipid droplets, a defining feature of adipocytes. These results confirm that AD-MSCs from peri-ovarian and peri-renal sites possess comparable multipotent differentiation capabilities, successfully committing to osteogenic, chondrogenic, and adipogenic lineages when provided with appropriate culture conditions (Figure 3).

### 3.4 Morphological changes during cardiomyocyte differentiation

**FIGURE 4:**
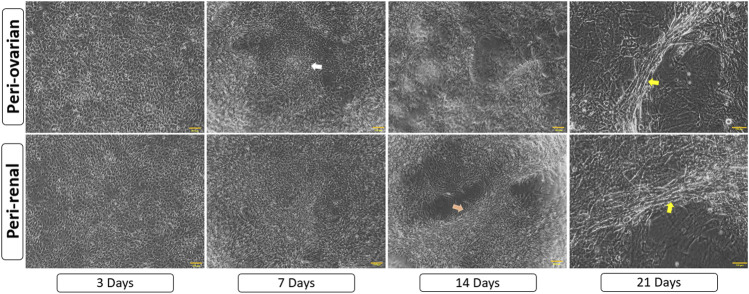
Morphological progression of AD-MSCs over 21 days following 5-azacytidine induction. Early spindle-shaped cells developed into colonies, showed intercellular connections, and later formed either clusters or myotube-like structures, indicating cardiomyocyte differentiation. Arrows highlight key features: white for cell aggregates/colonies, orange for aligned cells/connections, and yellow for myotube-like structures. Scale bar: 100 μm.

Three days after exposure to 5-azacytidine, AD-MSCs derived from both peri-ovarian and peri-renal adipose tissues exhibited circular or short spindle-shaped morphologies with single nuclei, characteristic of undifferentiated mesenchymal cells in primary cultures. By the first week, some adherent cells had undergone apoptosis, while the surviving cells proliferated and extended cytoplasmic processes toward adjacent cells. As differentiation progressed, the cells aggregated, increased in size, and formed small colonies. By the second week, adherent cells became aligned in a uniform direction, establishing extensive intercellular connections. After 3 weeks, distinct morphological changes were observed in both groups. Some cells formed spherical, ball-like structures, which may indicate early-stage cellular condensation, while others adopted elongated, myotube-like morphologies characteristic of cardiomyocyte differentiation. These morphological changes, along with representative structures, are indicated in Figure 4. The observed changes were consistent across AD-MSCs from both peri-ovarian and peri-renal sources. Based on morphological assessment, both types of AD-MSCs demonstrated similar responses to 5-azacytidine, with no significant differences in their cardiomyocyte differentiation potential.

### 3.5 Immunofluorescence staining

The expression of the cTnT was evaluated through immunofluorescence staining to confirm the cardiomyocyte differentiation of AD-MSCs. As expected, undifferentiated MSCs from the control groups, derived from both peri-ovarian and peri-renal adipose tissue, showed no detectable expression of cTnT, confirming their undifferentiated state.

In contrast, AD-MSCs from the induced groups (peri-ovarian and peri-renal), treated with 5-azacytidine, demonstrated a strong positive signal for cTnT 3 weeks post-induction. This finding highlights the successful activation of cardiac-specific protein expression, indicating differentiation toward a cardiomyocyte phenotype.

**FIGURE 5:**
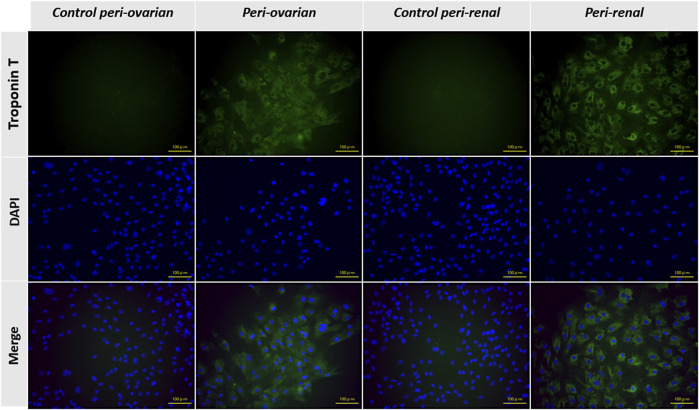
Representative immunofluorescence images showing the expression of cardiac troponin T (cTnT) in AD-MSCs across experimental groups. Undifferentiated cells from the control groups (peri-ovarian and peri-renal) displayed no detectable cTnT expression, confirming their non-cardiomyogenic phenotype. In contrast, AD-MSCs from the 5-azacytidine-induced groups (peri-ovarian and peri-renal) exhibited strong positive staining for cTnT 3 weeks post-induction, indicating successful differentiation toward a cardiomyocyte-like phenotype. Scale bar: 100 μm.

The positive immunofluorescent staining for cTnT in both induced groups provides compelling evidence of the efficacy of 5-azacytidine in directing AD-MSCs from both anatomical sites toward cardiomyocyte-like cells. Furthermore, the uniformity of cTnT expression across the two groups underscores the comparable differentiation potential of AD-MSCs isolated from these distinct adipose depots (Figure 5).

### 3.6 Metabolomic changes

#### 3.6.1 Metabolomic profiling of peri-ovarian and peri-renal AD-MSCs

Metabolomic analysis using GC-MS identified significant differences between the control and induced groups from both peri-ovarian and peri-renal AD-MSCs. A total of 112 metabolic features were detected, and 87 of these were found to be significantly altered across the groups. Comprehensive details of the significant findings among the studied groups are provided in Supplementary Tables S1, S2.

**FIGURE 6:**
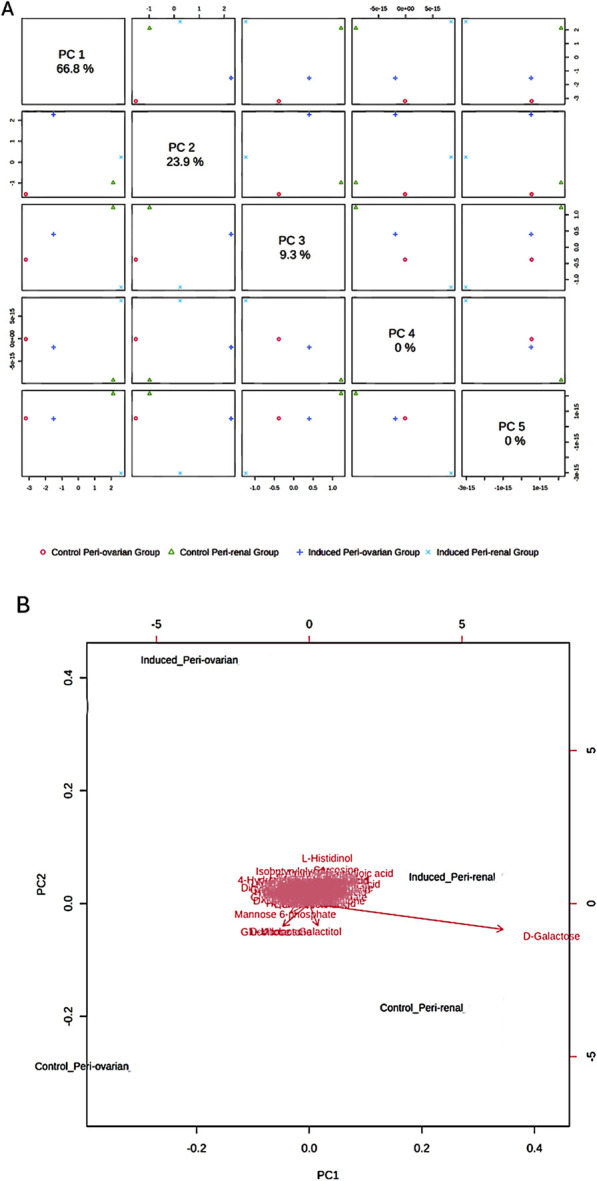
Principal Component Analysis (PCA) of Metabolomic Profiles. **(A)** Score Plot: A 2D score plot visualizing the clustering of the studied groups based on their metabolic profiles. The X-axis (PC1) and Y-axis (PC2) represent the first and second principal components, respectively, which explain 90.7% of the total variance in the data. PC1 accounts for the largest differences between the groups, particularly between the induced peri-ovarian and peri-renal AD-MSCs. While PC3, PC4, and PC5 were also calculated, they explain a much smaller proportion of the variance and were not included in the plot to maintain clarity. **(B)** Biplot: A biplot combining the score plot and loading plot, providing insights into the metabolites contributing to the group separation. The arrows represent the loadings (contributions) of individual metabolites to the principal components.

Principal Component Analysis (PCA) revealed distinct metabolic profiles among groups, with PC1 (66.8% variance) and PC2 (23.9% variance) explaining 90.7% of the total variance (Figure 6A). PC1 separated peri-ovarian AD-MSCs enriched in glycolysis and TCA cycle metabolites (e.g., Citric Acid, Glycerol) from peri-renal AD-MSCs, which showed higher reliance on D-Galactose. PC2 further differentiated induced groups from controls, underscoring metabolic shifts during differentiation (Figure 6B).

Induced peri-ovarian and peri-renal groups displayed distinct metabolic profiles compared to their control counterparts, indicating notable changes during cardiomyocyte differentiation. The PCA biplot further indicated that key metabolites, such as D-Galactose, L-Histidinol, and D-Ribose, contributed significantly to the separation of the groups. D-Galactose was the primary metabolite driving the separation between induced and control groups, suggesting its potential role in differentiation (Figure 6B).

#### 3.6.2 Identification of key metabolites driving cardiomyocyte differentiation

**FIGURE 7:**
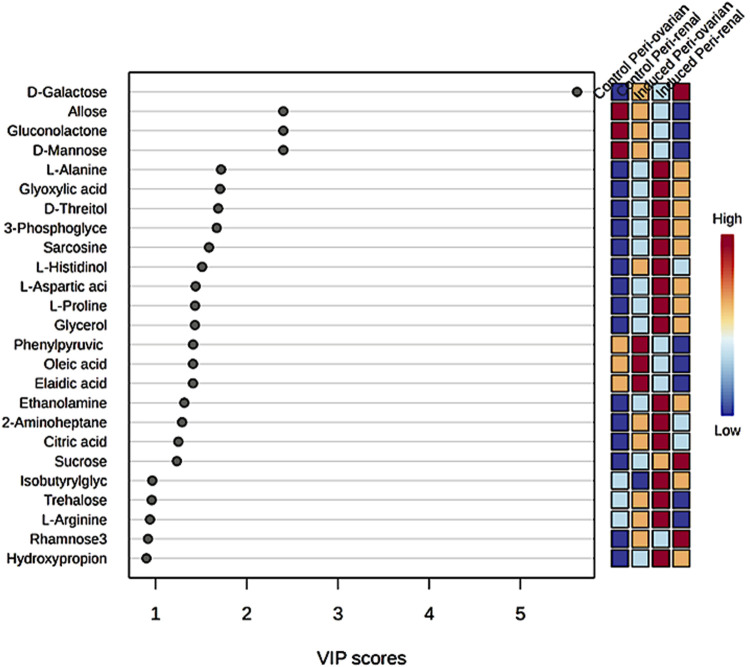
Partial Least Squares Discriminant Analysis (PLS-DA) illustrating the relative concentrations of the top 25 metabolites in each group under study.

Partial Least Squares Discriminant Analysis (PLS-DA) was performed to identify the top 25 metabolites contributing to group separation (Figure 7). The analysis revealed that D-Galactose was more abundant in the induced peri-renal group compared to the peri-ovarian group. In contrast, other metabolites such as D-Glucose, Fructose 1-phosphate, L-Histidinol, Glycerol, Ethanolamine, L-Proline, and Citric acid were significantly elevated in the induced peri-ovarian group relative to the peri-renal group.

These metabolites are essential for energy production and cellular differentiation. For example, Glycerol and Ethanolamine are involved in glycerolipid metabolism and phospholipid biosynthesis, crucial for membrane formation during differentiation, while Citric acid plays a key role in the TCA cycle, which is essential for ATP production and overall cellular energy metabolism. Additionally, D-Glucose and Fructose 1-phosphate are central to glycolysis and fructose metabolism, which contribute to energy production necessary for cardiomyocyte differentiation.

#### 3.6.3 Heatmap and pathway enrichment analysis

**FIGURE 8:**
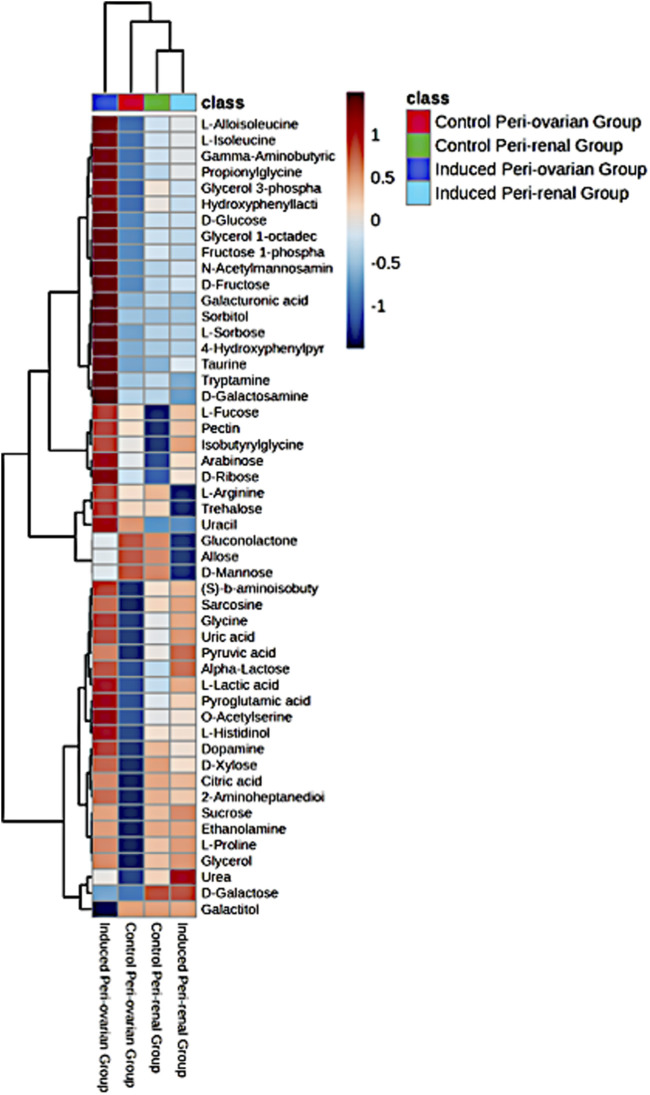
Heatmap representing the relative abundance of the top 25 significant metabolites across the four studied groups: Control Peri-ovarian, Induced Peri-ovarian, Control Peri-renal, and Induced Peri-renal. The color intensity indicates the relative level of each metabolite, with red representing higher abundance and blue representing lower abundance. The dendrogram on the left and top sides shows the hierarchical clustering of metabolites and groups, respectively.

A heatmap of the top 25 significant metabolites showed substantial differences between the groups, particularly between induced peri-ovarian and peri-renal AD-MSCs (Figure 8). Metabolites such as L-Histidinol, Glycerol, and Ethanolamine were highly elevated in the induced peri-ovarian group, suggesting a more active metabolic state conducive to cardiomyocyte differentiation. The presence of active metabolites in the control peri-renal group reflects the baseline metabolic activity of undifferentiated AD-MSCs, which is associated with cell maintenance, proliferation, and basic cellular functions. In contrast, the induced peri-renal group shows a shift in metabolic pathways, with a greater reliance on galactose metabolism as an alternative energy source. This shift may explain why fewer metabolites from the induced peri-renal group appear in the top 25 metabolites highlighted in the heatmap. It does not indicate a lack of metabolic activity but rather a different metabolic strategy compared to peri-ovarian AD-MSCs, which exhibit broader engagement of pathways like glycolysis and the TCA cycle.

To clarify the directionality of changes, we classified metabolites based on whether they were upregulated or downregulated during differentiation. In the induced peri-ovarian group, key metabolites such as D-glucose, Fructose 1-phosphate, Citric acid, Glycerol, Ethanolamine, and L-Proline were significantly upregulated, indicating enhanced glycolysis, TCA cycle activity, and lipid metabolism. In contrast, control peri-ovarian cells showed lower levels of these metabolites, reflecting a baseline metabolic state. In the induced peri-renal group, D-Galactose was notably upregulated, suggesting a shift toward galactose metabolism, whereas many glycolytic and TCA-related metabolites were either unchanged or downregulated compared to the peri-ovarian group. These distinctions underscore the metabolic strategies employed by each cell source during differentiation.

**FIGURE 9:**
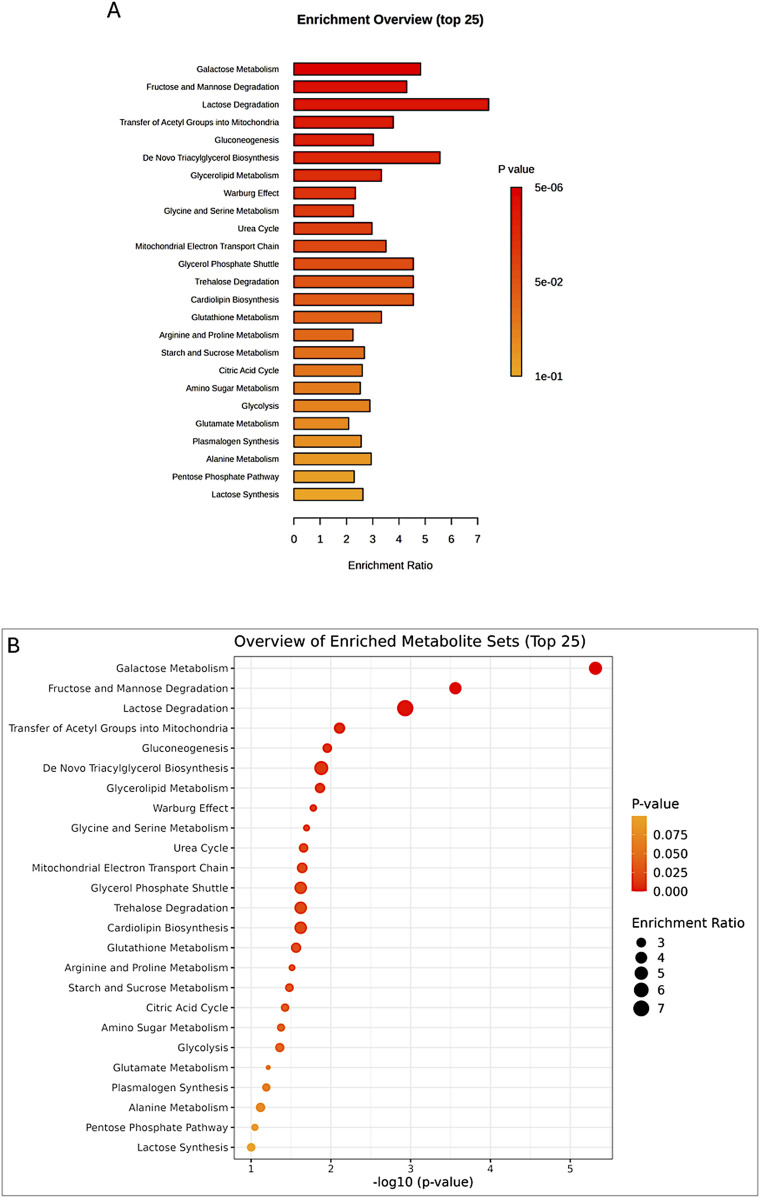
Enrichment analysis of significant metabolites, showing the top 25 pathways **(A,B)** potentially involved in the observed changes, based on the SMPDB Pathway library.

The enrichment analysis was performed on all four groups: control peri-ovarian, induced peri-ovarian, control peri-renal, and induced peri-renal. This approach allowed us to identify the metabolic pathways that are significantly enriched across these groups, with a particular focus on understanding how the metabolic landscape changes during cardiomyocyte differentiation and how it differs between the two anatomical sources (peri-ovarian vs peri-renal). The analysis revealed shared pathways, such as glycolysis and fructose metabolism, as well as distinct pathways, such as galactose metabolism in peri-renal AD-MSCs, that are activated during differentiation (Figure 9; Table 1).

**TABLE 1:** Enrichment analysis of significant metabolites, highlighting the top pathways potentially involved in group differences, based on the SMPDB Pathway metabolite set library.

Pathway	Total	Expected	Hits	Raw p	FDR
Galactose Metabolism	38	2.28	11	4.82E-06	0.00047
Fructose and Mannose Degradation	31	1.86	8	0.00028	0.0135
Lactose Degradation	9	0.539	4	0.00117	0.0382
Transfer of Acetyl Groups into Mitochondria	22	1.32	5	0.00779	0.167
Gluconeogenesis	33	1.98	6	0.0111	0.167
*De Novo* Triacylglycerol Biosynthesis	9	0.539	3	0.0132	0.167
Glycerolipid Metabolism	25	1.5	5	0.0137	0.167
Warburg Effect	57	3.41	8	0.0166	0.167
Glycine and Serine Metabolism	59	3.53	8	0.0202	0.167
Urea Cycle	28	1.68	5	0.022	0.167
Mitochondrial Electron Transport Chain	19	1.14	4	0.0229	0.167
Glycerol Phosphate Shuttle	11	0.659	3	0.0239	0.167
Trehalose Degradation	11	0.659	3	0.0239	0.167
Cardiolipin Biosynthesis	11	0.659	3	0.0239	0.167
Glutathione Metabolism	20	1.2	4	0.0273	0.179
Arginine and Proline Metabolism	52	3.11	7	0.0307	0.188
Starch and Sucrose Metabolism	31	1.86	5	0.0331	0.191
Citric Acid Cycle	32	1.92	5	0.0375	0.204
Amino Sugar Metabolism	33	1.98	5	0.0422	0.214
Glycolysis	23	1.38	4	0.0437	0.214
Glutamate Metabolism	48	2.87	6	0.061	0.285
Plasmalogen Synthesis	26	1.56	4	0.0645	0.287
Alanine Metabolism	17	1.02	3	0.0762	0.325
Pentose Phosphate Pathway	29	1.74	4	0.0897	0.366
Lactose Synthesis	19	1.14	3	0.0998	0.391
Carnitine Synthesis	22	1.32	3	0.14	0.527
Taurine and Hypotaurine Metabolism	12	0.719	2	0.158	0.575
Glucose-Alanine Cycle	13	0.778	2	0.18	0.631
Cysteine Metabolism	26	1.56	3	0.2	0.676
Propanoate Metabolism	42	2.51	4	0.24	0.758
Methionine Metabolism	42	2.51	4	0.24	0.758
Phospholipid Biosynthesis	29	1.74	3	0.249	0.762
Inositol Metabolism	30	1.8	3	0.265	0.768
Phosphatidylinositol Phosphate Metabolism	17	1.02	2	0.271	0.768
Valine, Leucine and Isoleucine Degradation	59	3.53	5	0.275	0.768
Ammonia Recycling	31	1.86	3	0.282	0.768
Spermidine and Spermine Biosynthesis	18	1.08	2	0.294	0.778
Pyruvate Metabolism	47	2.81	4	0.308	0.796
Catecholamine Biosynthesis	20	1.2	2	0.339	0.837
Aspartate Metabolism	35	2.1	3	0.35	0.837
Fatty Acid Biosynthesis	35	2.1	3	0.35	0.837
Inositol Phosphate Metabolism	24	1.44	2	0.427	0.938
Thiamine Metabolism	9	0.539	1	0.428	0.938
Homocysteine Degradation	9	0.539	1	0.428	0.938
Sphingolipid Metabolism	40	2.4	3	0.434	0.938
Malate-Aspartate Shuttle	10	0.599	1	0.462	0.938
Pyruvaldehyde Degradation	10	0.599	1	0.462	0.938
Oxidation of Branched Chain Fatty Acids	26	1.56	2	0.469	0.938
Phytanic Acid Peroxisomal Oxidation	26	1.56	2	0.469	0.938
Phenylalanine and Tyrosine Metabolism	27	1.62	2	0.489	0.951
D-Arginine and D-Ornithine Metabolism	11	0.659	1	0.495	0.951
Phosphatidylethanolamine Biosynthesis	12	0.719	1	0.525	0.99
Ketone Body Metabolism	13	0.778	1	0.554	1
Phosphatidylcholine Biosynthesis	14	0.838	1	0.581	1
Beta Oxidation of Very Long Chain Fatty Acids	17	1.02	1	0.653	1
Pyrimidine Metabolism	57	3.41	3	0.679	1
Vitamin B6 Metabolism	19	1.14	1	0.694	1
Butyrate Metabolism	19	1.14	1	0.694	1
Nucleotide Sugars Metabolism	20	1.2	1	0.713	1
Riboflavin Metabolism	20	1.2	1	0.713	1
Threonine and 2-Oxobutanoate Degradation	20	1.2	1	0.713	1
Pantothenate and CoA Biosynthesis	21	1.26	1	0.73	1
Betaine Metabolism	21	1.26	1	0.73	1
Sulfate/Sulfite Metabolism	22	1.32	1	0.747	1
Bile Acid Biosynthesis	65	3.89	3	0.764	1
Steroid Biosynthesis	48	2.87	2	0.798	1
Tyrosine Metabolism	70	4.19	3	0.808	1
Selenoamino Acid Metabolism	27	1.62	1	0.815	1
Mitochondrial Beta-Oxidation of Short Chain Saturated Fatty Acids	27	1.62	1	0.815	1
Mitochondrial Beta-Oxidation of Medium Chain Saturated Fatty Acids	27	1.62	1	0.815	1
Mitochondrial Beta-Oxidation of Long Chain Saturated Fatty Acids	28	1.68	1	0.827	1
Purine Metabolism	73	4.37	3	0.831	1
Folate Metabolism	29	1.74	1	0.837	1
Beta-Alanine Metabolism	34	2.04	1	0.882	1
Tryptophan Metabolism	59	3.53	2	0.883	1
Nicotinate and Nicotinamide Metabolism	35	2.1	1	0.889	1
Fatty Acid Elongation In Mitochondria	35	2.1	1	0.889	1
Porphyrin Metabolism	40	2.4	1	0.92	1
Histidine Metabolism	42	2.51	1	0.929	1
Fatty acid Metabolism	43	2.57	1	0.934	1

The broader metabolic reprogramming observed in peri-ovarian AD-MSCs is inferred from the integration of metabolite data (PLS-DA analysis and heatmap) with the pathway enrichment analysis (Table 1). Peri-ovarian AD-MSCs show higher levels of metabolites associated with glycolysis (e.g., D-Glucose, Fructose 1-phosphate), the TCA cycle (e.g., Citric acid), and glycerolipid metabolism (e.g., Glycerol, Ethanolamine), indicating engagement of multiple metabolic pathways. In contrast, peri-renal AD-MSCs exhibit higher levels of D-Galactose, suggesting a greater reliance on galactose metabolism. These findings collectively suggest that peri-ovarian AD-MSCs exhibit greater metabolic flexibility, engaging a wider range of pathways to support differentiation, while peri-renal AD-MSCs rely more heavily on galactose metabolism as an alternative energy source.

#### 3.6.4 Chemical structure-based enrichment analysis

**FIGURE 10:**
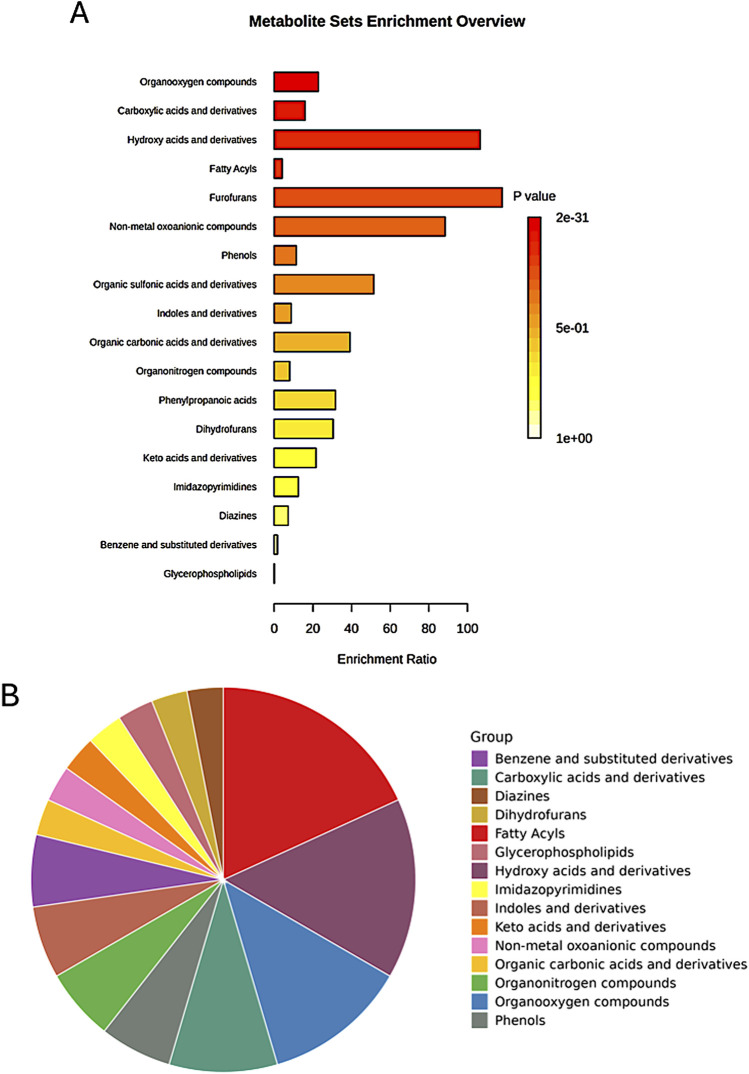
Enrichment analysis of significant metabolites based on the metabolite set library of chemical structures, highlighting the top involved structures for the main classes among the studied groups. **(A)** Bar chart displaying the top structures; **(B)** Pie chart summarizing the distribution of the main classes.

The enrichment analysis extended beyond pathway involvement to encompass the chemical structures of the identified metabolites. This analysis classified metabolites based on their main and sub-classes, revealing the predominant chemical structures associated with the observed metabolic changes (Figure 10).

Among the sub-classes, organooxygen compounds emerged as the most enriched, suggesting a significant role for oxygen-containing molecules in the metabolic processes differentiating the studied groups (Table 2). Carboxylic acids and their derivatives also exhibited substantial enrichment, highlighting the importance of these organic acids in the metabolic landscape. Other enriched sub-classes included hydroxy acids and derivatives, fatty acyls, and non-metal oxoanionic compounds, each contributing to the unique metabolic signatures of the groups.

**TABLE 2:** Enrichment analysis of significant features among the groups, based on the metabolite set library of chemical structures (sub-classes), highlighting the top involved structures.

Chemical structure	Total	Expected	Hits	Raw p	Holm p	FDR
Organooxygen compounds	3160	1.27	29	2.08E-31	9.88E-29	9.88E-29
Carboxylic acids and derivatives	3740	1.51	24	2.87E-22	1.36E-19	6.83E-20
Hydroxy acids and derivatives	116	0.0469	5	1.48E-09	7.00E-07	2.34E-07
Fatty Acyls	4680	1.89	8	0.00063	0.298	0.075
Furofurans	21	0.00848	1	0.00845	1	0.804
Non-metal oxoanionic compounds	28	0.0113	1	0.0113	1	0.893
Phenols	434	0.175	2	0.0135	1	0.92
Organic sulfonic acids and derivatives	48	0.0194	1	0.0192	1	1
Indoles and derivatives	559	0.226	2	0.0218	1	1
Organic carbonic acids and derivatives	63	0.0255	1	0.0251	1	1
Organonitrogen compounds	618	0.25	2	0.0262	1	1
Phenylpropanoic acids	78	0.0315	1	0.031	1	1
Dihydrofurans	81	0.0327	1	0.0322	1	1
Keto acids and derivatives	114	0.0461	1	0.045	1	1
Imidazopyrimidines	198	0.08	1	0.0769	1	1
Diazines	342	0.138	1	0.129	1	1
Benzene and substituted derivatives	3050	1.23	2	0.35	1	1
Glycerophospholipids	1560	16.2	1	1	1	1

Specific metabolites within these enriched sub-classes further illuminated the metabolic distinctions. Fatty acyls, for instance, encompassed key molecules like palmitic acid, dodecanoic acid, and myristic acid, suggesting alterations in fatty acid metabolism (Table 3). Hydroxy acids and derivatives included metabolites such as malic acid and lactic acid, pointing to potential shifts in energy metabolism and related pathways. The enrichment of organooxygen compounds encompassed a wide array of metabolites, including sugars, sugar derivatives, and oxygen-containing amino acids, underscoring the diverse roles of oxygen-containing molecules in cellular processes. Overall, the chemical structure-based enrichment analysis provided a complementary perspective to the pathway analysis, emphasizing the specific chemical classes and metabolites driving the metabolic differences between the groups. These findings suggest that oxygen-containing molecules, organic acids, fatty acids, and hydroxy acids play pivotal roles in the metabolic processes distinguishing the studied groups, offering potential targets for further investigation and therapeutic intervention.

**TABLE 3:** Top chemical structures identified from the enrichment analysis of significant features among the groups, based on the metabolite set library of chemical structures (sub-classes).

Group	Hits	Members
Fatty Acyls	8	Palmitic acid; Dodecanoic acid; Octanoylcarnitine; Myristic acid; Stearic acid; Pimelic acid; Heptadecanoic acid; Octadecanol
Hydroxy acids and derivatives	5	2-Hydroxybutyric acid; Malic acid; Lactic acid; Hydroxypropionic acid; 3-Dehydroquinic acid
Organooxygen compounds	29	D-Xylose; Galactitol; D-Glucose; D-Glucuronic acid; Glycerol; D-Galactose; Gluconolactone; D-Maltose; D-Mannose; L-Fucose; Alpha-Lactose; myo-Inositol; Sorbitol; Sucrose; D-Ribose; L-Arabinose; D-Fructose; Mannitol; Trehalose; Fructose 1-phosphate; Mannose 6-phosphate; N-Acetylmannosamine; Allose; L-Sorbose; Dihydroxyacetone phosphate; Glucosamine; Galacturonic acid; Pectin; D-Tagatose
Carboxylic acids and derivatives	24	Citric acid; gamma-Aminobutyric acid; Glycine; Phenylalanine; Proline; L-Threonine; Isoleucine; Ornithine; Succinic acid; Pyroglutamic acid; Sarcosine; L-Arginine; L-Alloisoleucine; L-Cysteine; Isobutyrylglycine; Propionylglycine; Ureidosuccinic acid; L-Valine; (S)-beta-Aminoisobutyric acid; Oxalic acid; O-Acetylserine; N2-Acetylornithine; N-Acetylglutamine; 2-Aminoheptanedioic acid
Organonitrogen compounds	2	Ethanolamine; L-Histidinol
Phenols	2	Dopamine; Coniferyl alcohol
Benzene and substituted derivatives	2	4-Hydroxyphenylpyruvic acid; Benzoic acid
Indoles and derivatives	2	Indoleacetic acid; Tryptamine
Keto acids and derivatives	1	Pyruvic acid
Organic carbonic acids and derivatives	1	Urea
Imidazopyrimidines	1	Uric acid
Dihydrofurans	1	Ascorbic acid
Diazines	1	Uracil
Glycerophospholipids	1	Glycerol 3-phosphate
Non-metal oxoanionic compounds	1	Phosphate

## 4 Discussion

MSCs exhibit rapid *in vitro* proliferation and the ability to differentiate into cells of both mesenchymal and non-mesenchymal origins. Traditionally, MSCs have been primarily sourced from bone marrow; however, the low cell yields and invasive harvest procedures associated with this method make it a less preferable cell source (Elnakish et al., 2012; Choudhery et al., 2015). Adipose-derived stem cells have emerged as a promising regenerative medicine resource, offering both biological advantages and practical benefits. Unlike pluripotent stem cell sources (embryonic or induced pluripotent stem cells), adipose tissue eliminates ethical controversies while mitigating risks of tumorigenesis and immune rejection (Fraser et al., 2006). From a translational perspective, adipose tissue demonstrates superior clinical utility as a progenitor cell source, yielding 100-500 times more viable stem cells per unit volume compared to conventional bone marrow aspirates (Casteilla et al., 2011). This makes it an important consideration to explore the potential capabilities of MSCs derived from adipose tissue, obtained from various anatomical locations.

To the best of our knowledge, this study is the first of its kind to comprehensively compare the cardiomyocyte differentiation potential of AD-MSCs derived from two distinct anatomical sites (peri-ovarian and peri-renal) using a combination of morphological, immunophenotypic, functional, and metabolomic analyses. While previous studies have explored the differentiation potential of AD-MSCs from various sources, none have specifically investigated the impact of anatomical origin on metabolic adaptations during cardiomyocyte differentiation. By integrating metabolomic profiling with traditional functional assays, this study provides novel insights into how the anatomical site influences the metabolic landscape of AD-MSCs, offering a new perspective on optimizing stem cell-based therapies for cardiac regeneration.

Both peri-ovarian and peri-renal AD-MSCs exhibited similar morphological characteristics, displaying the typical fibroblast-like morphology of mesenchymal stem cells. This consistency in morphology suggests that the anatomical origin does not significantly affect the basic cellular structure of AD-MSCs. Immunophenotypic analysis via flow cytometry further confirmed that both groups expressed the characteristic MSC markers CD44, CD90, and CD29, while lacking expression of the hematopoietic marker CD45. Additionally, both AD-MSC populations demonstrated trilineage differentiation potential, successfully differentiating into osteogenic, chondrogenic, and adipogenic lineages under specific induction conditions. These findings align with previous studies demonstrating the reliability of these markers and the multipotent nature of MSCs, regardless of their anatomical origin (Yoshimura et al., 2007; Kaewkhaw et al., 2011; Jia et al., 2015; Li et al., 2015; Pittenger et al., 2019; Hendawy et al., 2021; Sun et al., 2021).

Following cardiomyocyte induction, both groups displayed similar morphological changes, including the formation of myotube-like structures, indicative of cardiomyogenic differentiation, as described in earlier studies (Makino et al., 1999; Eldin et al., 2019). Immunofluorescence staining for cardiac troponin T (cTnT) confirmed successful differentiation in both groups, with comparable fluorescence intensity observed in peri-ovarian and peri-renal AD-MSCs, These findings are in agreement with prior studies (Van Dijk et al., 2008; Xing et al., 2012b). These results suggest that the anatomical origin does not significantly impact the qualitative potential for cardiomyocyte differentiation, as assessed by morphology and IF.

While the morphological, IF, and FCM results showed no significant differences between the two AD-MSC sources, the metabolomic analysis revealed distinct metabolic adaptations. Peri-ovarian AD-MSCs exhibited broader metabolic reprogramming, engaging pathways such as glycolysis, TCA cycle, and glycerolipid metabolism, while peri-renal AD-MSCs relied more heavily on galactose metabolism. These metabolic differences suggest that while the two groups exhibit similar morphological, functional, and multilineage differentiation characteristics, they employ different metabolic strategies to support differentiation. This highlights the importance of considering both functional and metabolic profiles when evaluating the differentiation potential of AD-MSCs from different anatomical sources.

The metabolomic analysis revealed distinct metabolic adaptations in AD-MSCs from peri-ovarian and peri-renal sources during cardiomyocyte differentiation. Both groups shared key metabolic pathways such as glycolysis, fructose metabolism, and glycerolipid metabolism, which are essential for energy production and biosynthesis (Yen et al., 2008; Vander Heiden et al., 2009; Lunt and Vander Heiden, 2011; Ito and Suda, 2014; Softic et al., 2017; Jang et al., 2018). However, peri-ovarian AD-MSCs exhibited broader metabolic reprogramming, with enhanced engagement of the TCA cycle and phospholipid biosynthesis, suggesting a more metabolically active state conducive to differentiation. These findings align with previous studies demonstrating the crucial role of TCA cycle intermediates and phospholipid metabolism in cardiomyocyte development and function (Stanley et al., 2005; Fillmore and Lopaschuk, 2013; Kolwicz Jr et al., 2013; van der Veen et al., 2017). We also observed that D-Galactose plays a dual role in this study, depending on the context of the comparison. In the control vs induced comparison, D-Galactose contributes to the metabolic reprogramming associated with cardiomyocyte differentiation. However, when comparing induced peri-renal and peri-ovarian AD-MSCs, D-Galactose is more abundant in the peri-renal group, suggesting that peri-renal AD-MSCs rely more heavily on galactose metabolism as an alternative energy source during differentiation. This dual role highlights the complexity of metabolic adaptations during differentiation and underscores the importance of considering anatomical origin when interpreting metabolomic data.

The observed differences in metabolite profiles between the groups suggest that peri-ovarian AD-MSCs rely more heavily on glycolysis, fructose metabolism, and the TCA cycle, reflecting a higher energy demand. In contrast, the peri-renal group exhibited a relative enrichment in galactose metabolism, suggesting an alternative energy source during differentiation. These metabolic distinctions highlight the site-specific characteristics of AD-MSCs, reinforcing the importance of considering tissue origin when optimizing stem cell-based therapies for cardiac regeneration (Fraser et al., 2006; Gimble et al., 2007; Pachón-Peña et al., 2011; Baer and Geiger, 2012; Shyh-Chang and Ng, 2017).

The TCA cycle plays a pivotal role in cardiomyocyte differentiation by providing the necessary energy and biosynthetic precursors for developing cardiomyocytes. During differentiation, the TCA cycle generates ATP through oxidative phosphorylation, which is essential for meeting the high energy demands of contracting cardiomyocytes (Kolwicz Jr et al., 2013). Additionally, TCA cycle intermediates such as citrate and α-ketoglutarate serve as precursors for the synthesis of amino acids, nucleotides, and lipids, which are critical for cellular growth and function (Fillmore and Lopaschuk, 2013). In our study, the elevated levels of Citric acid in peri-ovarian AD-MSCs suggest enhanced TCA cycle activity, supporting the idea that these cells engage in robust energy production and biosynthetic processes during differentiation. This aligns with previous studies showing that TCA cycle activation is essential for the maturation and functional development of cardiomyocytes (Lopaschuk and Jaswal, 2010).

Glycolysis is a fundamental metabolic pathway that provides ATP and intermediates for biosynthetic processes, particularly under conditions of high energy demand. During cardiomyocyte differentiation, glycolysis supports the rapid proliferation and maturation of cells by supplying pyruvate for the TCA cycle and generating intermediates for nucleotide and amino acid synthesis (Vander Heiden et al., 2009). The elevated levels of D-Glucose and Fructose 1-phosphate in peri-ovarian AD-MSCs indicate active glycolysis, which is consistent with the increased energy requirements of differentiating cells.

Fructose metabolism serves as an alternative pathway for energy production and biosynthesis, complementing glycolysis and the TCA cycle. Fructose is metabolized to fructose-1-phosphate, which enters glycolysis and provides intermediates for biosynthetic pathways (Softic et al., 2017). In peri-ovarian AD-MSCs, the elevated levels of Fructose 1-phosphate suggest that fructose metabolism contributes to energy production and supports the synthesis of cellular components during differentiation. Fructose metabolism may also play a role in modulating oxidative stress and protecting cells from damage during differentiation (Jang et al., 2018). This pathway is particularly important in tissues with high energy demands, such as the heart, and its activation in peri-ovarian AD-MSCs underscores its relevance in cardiomyocyte differentiation.

Galactose metabolism represents an alternative energy strategy, particularly in peri-renal AD-MSCs. Galactose is converted to glucose-6-phosphate through the Leloir pathway, which can then enter glycolysis or the pentose phosphate pathway to generate ATP and biosynthetic precursors (Coelho et al., 2017). The elevated levels of D-Galactose in the induced peri-renal group suggest that these cells rely on galactose metabolism to meet their energy needs during differentiation. This pathway may provide a metabolic advantage under conditions where glucose availability is limited or when alternative energy sources are required.

The upregulation of galactose metabolism observed in peri-renal AD-MSCs suggests a strategic metabolic adaptation during cardiomyocyte differentiation. Unlike glucose, galactose metabolism does not support high glycolytic flux and instead promotes a shift toward mitochondrial oxidative phosphorylation (OXPHOS), which is essential for the maturation of cardiomyocytes. This transition is critical, as developing cardiomyocytes demand sustained ATP production to support biosynthesis, contractile function, and structural remodeling. Notably, metabolic reprogramming such as the HIF1α-mediated shift to glycolysis has been shown to suppress mitochondrial respiration and is associated with less mature stem cell states (Zhou et al., 2012), underscoring the importance of OXPHOS in driving differentiation and functional maturation.

In addition, cells cultured in galactose-supplemented media exhibit increased mitochondrial biogenesis and respiratory chain activity, reflecting a more oxidative metabolic phenotype (Rossignol et al., 2004). Thus, the reliance on galactose metabolism may signify that peri-renal AD-MSCs engage a more energetically efficient differentiation program under certain microenvironmental cues. While peri-ovarian AD-MSCs utilize glycolysis and the TCA cycle more broadly, peri-renal cells may be metabolically primed for efficient ATP generation through oxidative metabolism, aligning with energy demands during late-stage cardiomyocyte differentiation. These findings are consistent with earlier reports indicating that mitochondrial biogenesis and a metabolic shift toward oxidative phosphorylation are hallmarks of stem cell differentiation and cardiomyocyte maturation (Wanet et al., 2015). Culture systems utilizing galactose, which force cells to rely more on oxidative metabolism, have been shown to enhance mitochondrial function and promote cardiomyocyte maturation.

Chemical structure-based enrichment analysis provided further insight into the metabolic landscape, revealing significant involvement of organooxygen compounds, carboxylic acids and derivatives, hydroxy acids, and fatty acyls. These molecular classes play key roles in lipid metabolism, energy homeostasis, and structural remodeling, all of which are critical for cellular differentiation. The enrichment of fatty acyls, including palmitic acid and dodecanoic acid, suggests an active role of lipid metabolism in membrane synthesis and signaling pathways during cardiomyogenesis (Listenberger et al., 2003; Glatz et al., 2010). Similarly, the presence of hydroxy acids, such as malic acid and lactic acid, underscores shifts in energy metabolism, potentially reflecting changes in oxidative and glycolytic balance during differentiation (Kolwicz Jr et al., 2013; Hui et al., 2017).

The findings of this study emphasize the intricate metabolic reprogramming required for cardiomyocyte differentiation and the potential influence of stem cell origin on metabolic pathways. The distinct metabolomic profiles of peri-ovarian and peri-renal AD-MSCs provide a foundation for future research exploring metabolic modulation as a strategy to enhance stem cell differentiation and therapeutic efficacy. Further investigations into how these metabolic pathways influence gene expression and functional cardiomyocyte development could refine the use of AD-MSCs in regenerative medicine.

Although peri-ovarian and peri-renal AD-MSCs demonstrated similar differentiation efficiency, the broader metabolic reprogramming observed in peri-ovarian AD-MSCs marked by upregulated glycolysis, TCA cycle, and glycerolipid metabolism suggests potential advantages for cardiac regeneration. For example, glycolytic metabolism enhances stem cell survival in hypoxic environments typical of ischemic myocardium (Luo et al., 2023), while efficient TCA cycle activity is critical for sustaining the high energetic demands of functional cardiomyocytes (Lopaschuk and Jaswal, 2010; Lopaschuk et al., 2021). In contrast, peri-renal AD-MSCs’ reliance on galactose metabolism, though adaptive, may limit their capacity to mitigate oxidative stress during engraftment (Hu et al., 2022; Xu et al., 2025). These metabolic distinctions could influence post-transplantation outcomes, positioning peri-ovarian AD-MSCs as a more promising candidate for cardiac repair.

Additionally, while both peri-ovarian and peri-renal AD-MSCs exhibited comparable cardiomyocyte differentiation (as evidenced by cTnT expression), the observed metabolic differences such as enhanced glycolysis/TCA cycle activity in peri-ovarian AD-MSCs *versus* reliance on galactose metabolism in peri-renal AD-MSCs may influence functional maturation of derived cardiomyocytes. For instance, glycolytic flux and TCA cycle efficiency are critical for calcium handling and contractility in mature cardiomyocytes (Lopaschuk and Jaswal, 2010; Kolwicz Jr et al., 2013), whereas alternative pathways like galactose metabolism may reflect adaptive energy strategies under metabolic stress (Coelho et al., 2015). Future studies should assess functional metrics (e.g., electrophysiology, contractile force, or mitochondrial respiration) to determine whether anatomical origin impacts the therapeutic efficacy of AD-MSC-derived cardiomyocytes in regenerative applications.

## 5 Limitations

Three key limitations should be considered. First, as an *in vitro* study, these results require validation in physiological *in vivo* models. Second, while our GC-MS metabolomics approach successfully identified pathway differences, this technique has inherent analytical constraints: (1) it cannot detect all metabolite classes equally (particularly large or unstable molecules), (2) sample preparation may artificially alter some metabolites, and (3) it measures whole-cell metabolite levels without revealing their subcellular distributions. Third, our single timepoint analysis provides a metabolic snapshot that may not capture dynamic changes during differentiation. Future studies combining complementary analytical techniques with temporal profiling could address these limitations.

## 6 Conclusion

This study provides novel insights into the cardiomyocyte differentiation potential of AD-MSCs harvested from peri-ovarian and peri-renal anatomical sites, utilizing a comprehensive approach combining morphological, immunophenotypic, functional, and metabolomic analyses. Both AD-MSC populations exhibited comparable morphological characteristics, immunophenotypic profiles, multilineage differentiation potential, and successful cardiomyocyte differentiation as evidenced by morphological changes and cTnT expression. Metabolomic profiling revealed distinct metabolic adaptations, with peri-ovarian AD-MSCs engaging a broader range of pathways including glycolysis, TCA cycle, and glycerolipid metabolism, while peri-renal AD-MSCs showed greater reliance on galactose metabolism. These findings demonstrate that anatomical origin influences metabolic strategies during differentiation.

While the broader metabolic flexibility observed in peri-ovarian AD-MSCs may suggest potential advantages for cardiac regeneration, given the established importance of these pathways in supporting cell survival and function, we emphasize that direct comparative studies of therapeutic efficacy are needed to determine if these metabolic differences translate to functional benefits *in vivo*. Future research should examine whether these distinct metabolic profiles correlate with differences in cardiomyocyte maturation, electrophysiological properties, or engraftment efficiency in preclinical models. These results highlight the value of metabolic profiling in understanding stem cell behavior and provide a foundation for optimizing cell source selection in regenerative applications.

## Data Availability

The datasets presented in this study can be found in online repositories. The names of the repository/repositories and accession number(s) can be found in the article/Supplementary Material.
