# Supplementary material for: Comparative cardiomyocyte differentiation potential of rat adipose-derived mesenchymal stem cells from two anatomical sites: metabolomic profiling and pathway analysis
Source: Front Cell Dev Biol. 2025 Jun 19;13:1604605. doi: 10.3389/fcell.2025.1604605 (PMC12222180; doi:10.3389/fcell.2025.1604605)
Supplement: Supplementary file 1 [file Table1.DOCX]

**Supplementary Material**

**Supplementary Table 1.** Significant features (metabolites) among the studied groups based on ANOVA.

| Features | F-stat | p. value | FDR |
| --- | --- | --- | --- |
| Trehalose | 9.45E+32 | 1.56E-130 | 1.36E-128 |
| D-Ribose | 1.62E+32 | 1.81E-127 | 7.88E-126 |
| Glycerol | 8.10E+31 | 2.89E-126 | 8.37E-125 |
| D-Xylose | 7.15E+31 | 4.75E-126 | 8.94E-125 |
| D-Fructose | 7.01E+31 | 5.14E-126 | 8.94E-125 |
| L-Histidinol | 5.37E+31 | 1.49E-125 | 2.16E-124 |
| L-Proline | 4.42E+31 | 3.26E-125 | 4.05E-124 |
| 2-Aminoheptanedioic acid | 3.08E+31 | 1.39E-124 | 1.51E-123 |
| Dopamine | 2.33E+31 | 4.24E-124 | 4.10E-123 |
| Citric acid | 2.06E+31 | 6.91E-124 | 6.01E-123 |
| Ethanolamine | 1.68E+31 | 1.55E-123 | 1.23E-122 |
| Uric acid | 1.60E+31 | 1.91E-123 | 1.38E-122 |
| D-Galactose | 1.24E+31 | 5.19E-123 | 3.47E-122 |
| Pectin | 7.09E+30 | 4.92E-122 | 2.86E-121 |
| L-Fucose | 7.09E+30 | 4.92E-122 | 2.86E-121 |
| Sorbitol | 5.72E+30 | 1.16E-121 | 6.33E-121 |
| Glycerol 1-octadecanoate | 5.24E+30 | 1.65E-121 | 8.46E-121 |
| L-Lactic acid | 4.94E+30 | 2.09E-121 | 1.01E-120 |
| Sucrose | 4.83E+30 | 2.29E-121 | 1.05E-120 |
| Hydroxyphenyllactic acid | 4.59E+30 | 2.80E-121 | 1.22E-120 |
| Galacturonic acid | 4.44E+30 | 3.22E-121 | 1.33E-120 |
| Glycine | 3.58E+30 | 7.61E-121 | 3.01E-120 |
| Allose | 3.38E+30 | 9.51E-121 | 3.31E-120 |
| Gluconolactone | 3.38E+30 | 9.51E-121 | 3.31E-120 |
| D-Mannose | 3.38E+30 | 9.51E-121 | 3.31E-120 |
| Taurine | 3.26E+30 | 1.10E-120 | 3.67E-120 |
| Fructose 1-phosphate | 3.15E+30 | 1.26E-120 | 4.02E-120 |
| Isobutyrylglycine | 3.13E+30 | 1.29E-120 | 4.02E-120 |
| Sarcosine | 2.40E+30 | 3.75E-120 | 1.13E-119 |
| L-Isoleucine | 2.11E+30 | 6.28E-120 | 1.71E-119 |
| L-Alloisoleucine | 2.11E+30 | 6.28E-120 | 1.71E-119 |
| Gamma-Aminobutyric acid | 2.11E+30 | 6.28E-120 | 1.71E-119 |
| D-Glucose | 1.48E+30 | 2.61E-119 | 6.88E-119 |
| L-Arginine | 1.43E+30 | 3.00E-119 | 7.68E-119 |
| Pyruvic acid | 1.41E+30 | 3.15E-119 | 7.83E-119 |
| O-Acetylserine | 1.40E+30 | 3.24E-119 | 7.83E-119 |
| Uracil | 1.26E+30 | 4.93E-119 | 1.16E-118 |
| 4-Hydroxyphenylpyruvic acid | 1.13E+30 | 7.67E-119 | 1.76E-118 |
| Pyroglutamic acid | 9.41E+29 | 1.59E-118 | 3.54E-118 |
| D-Galactosamine | 9.23E+29 | 1.72E-118 | 3.73E-118 |
| Urea | 7.28E+29 | 4.43E-118 | 9.26E-118 |
| L-Sorbose | 7.26E+29 | 4.47E-118 | 9.26E-118 |
| Glycerol 3-phosphate | 7.05E+29 | 5.04E-118 | 1.02E-117 |
| Alpha-Lactose | 6.58E+29 | 6.64E-118 | 1.31E-117 |
| Galactitol | 6.06E+29 | 9.20E-118 | 1.78E-117 |
| N-Acetylmannosamine | 5.13E+29 | 1.80E-117 | 3.40E-117 |
| Arabinose | 4.91E+29 | 2.15E-117 | 3.98E-117 |
| Propionylglycine | 4.73E+29 | 2.49E-117 | 4.51E-117 |
| Tryptamine | 4.19E+29 | 4.03E-117 | 6.99E-117 |
| (S)-b-aminoisobutyric acid | 4.17E+29 | 4.10E-117 | 6.99E-117 |
| L-Valine | 4.17E+29 | 4.10E-117 | 6.99E-117 |
| Oxalic acid | 3.92E+29 | 5.25E-117 | 8.46E-117 |
| 2-Hydroxybutyric acid | 3.92E+29 | 5.25E-117 | 8.46E-117 |
| Hydroxypropionic acid | 3.92E+29 | 5.25E-117 | 8.46E-117 |
| Ornithine | 3.58E+29 | 7.60E-117 | 1.20E-116 |
| Mannose 6-phosphate | 3.29E+29 | 1.06E-116 | 1.65E-116 |
| Heptadecanoic acid | 2.89E+29 | 1.78E-116 | 2.66E-116 |
| Octadecanol | 2.89E+29 | 1.78E-116 | 2.66E-116 |
| 3-Dehydroquinate | 2.51E+29 | 3.12E-116 | 4.60E-116 |
| D-Glucuronic acid | 2.35E+29 | 4.06E-116 | 5.89E-116 |
| Coniferyl aldehyde | 1.96E+29 | 8.36E-116 | 1.19E-115 |
| L-Octanoylcarnitine | 1.93E+29 | 8.93E-116 | 1.25E-115 |
| D-Maltose | 1.84E+29 | 1.09E-115 | 1.50E-115 |
| L-Malic acid | 1.75E+29 | 1.33E-115 | 1.80E-115 |
| Dihydroxyacetone phosphate | 1.75E+29 | 1.34E-115 | 1.80E-115 |
| D-Tagatose | 1.74E+29 | 1.36E-115 | 1.80E-115 |
| L-Threonine | 1.46E+29 | 2.75E-115 | 3.58E-115 |
| L-Phenylalanine | 1.25E+29 | 5.08E-115 | 6.49E-115 |
| N-Acetylornithine | 1.02E+29 | 1.17E-114 | 1.48E-114 |
| Benzoic acid | 7.62E+28 | 3.69E-114 | 4.59E-114 |
| L-Cysteine | 5.99E+28 | 9.64E-114 | 1.18E-113 |
| Myoinositol | 5.94E+28 | 1.00E-113 | 1.21E-113 |
| Indoleacetic acid | 4.99E+28 | 2.01E-113 | 2.39E-113 |
| Palmitic acid | 4.94E+28 | 2.09E-113 | 2.46E-113 |
| Mannitol | 4.39E+28 | 3.36E-113 | 3.90E-113 |
| N-Acetylglutamine | 3.85E+28 | 5.65E-113 | 6.46E-113 |
| Ascorbic acid | 3.17E+28 | 1.23E-112 | 1.39E-112 |
| Coniferyl alcohol | 2.69E+28 | 2.38E-112 | 2.66E-112 |
| Ureidosuccinic acid | 1.99E+28 | 7.94E-112 | 8.74E-112 |
| Succinic acid | 1.76E+28 | 1.30E-111 | 1.41E-111 |
| Glucosamine | 1.14E+28 | 7.49E-111 | 8.05E-111 |
| D-Glucurono-6,3-lactone | 8.28E+27 | 2.65E-110 | 2.81E-110 |
| Dodecanoic acid | 7.49E+27 | 3.95E-110 | 4.14E-110 |
| Stearic acid | 7.21E+27 | 4.60E-110 | 4.76E-110 |
| Phosphoric acid | 1.55E+27 | 2.16E-107 | 2.21E-107 |
| Pimelic acid | 1.37E+27 | 3.58E-107 | 3.62E-107 |
| Myristic acid | 8.40E+26 | 2.49E-106 | 2.49E-106 |

**Supplementary Table 2.** Significant metabolites among the studied groups based on ANOVA and Fisher LSD test.

| Features | F. value | p. value | FDR | Fisher's LSD |
| --- | --- | --- | --- | --- |
| Trehalose | 9.45E+32 | 1.56E-130 | 1.36E-128 | Control Peri-renal Group - Control Peri-ovarian Group; Induced Peri-ovarian Group - Control Peri-ovarian Group; Control Peri-ovarian Group - Induced Peri-renal Group; Induced Peri-ovarian Group - Control Peri-renal Group; Control Peri-renal Group - Induced Peri-renal Group; Induced Peri-ovarian Group - Induced Peri-renal Group |
| D-Ribose | 1.62E+32 | 1.81E-127 | 7.88E-126 | Control Peri-ovarian Group - Control Peri-renal Group; Induced Peri-ovarian Group - Control Peri-ovarian Group; Induced Peri-renal Group - Control Peri-ovarian Group; Induced Peri-ovarian Group - Control Peri-renal Group; Induced Peri-renal Group - Control Peri-renal Group; Induced Peri-ovarian Group - Induced Peri-renal Group |
| Glycerol | 8.10E+31 | 2.89E-126 | 8.37E-125 | Control Peri-renal Group - Control Peri-ovarian Group; Induced Peri-ovarian Group - Control Peri-ovarian Group; Induced Peri-renal Group - Control Peri-ovarian Group; Induced Peri-ovarian Group - Control Peri-renal Group; Induced Peri-renal Group - Control Peri-renal Group; Induced Peri-ovarian Group - Induced Peri-renal Group |
| D-Xylose | 7.15E+31 | 4.75E-126 | 8.94E-125 | Control Peri-renal Group - Control Peri-ovarian Group; Induced Peri-ovarian Group - Control Peri-ovarian Group; Induced Peri-renal Group - Control Peri-ovarian Group; Induced Peri-ovarian Group - Control Peri-renal Group; Control Peri-renal Group - Induced Peri-renal Group; Induced Peri-ovarian Group - Induced Peri-renal Group |
| D-Fructose | 7.01E+31 | 5.14E-126 | 8.94E-125 | Control Peri-renal Group - Control Peri-ovarian Group; Induced Peri-ovarian Group - Control Peri-ovarian Group; Induced Peri-renal Group - Control Peri-ovarian Group; Induced Peri-ovarian Group - Control Peri-renal Group; Induced Peri-renal Group - Control Peri-renal Group; Induced Peri-ovarian Group - Induced Peri-renal Group |
| L-Histidinol | 5.37E+31 | 1.49E-125 | 2.16E-124 | Control Peri-renal Group - Control Peri-ovarian Group; Induced Peri-ovarian Group - Control Peri-ovarian Group; Induced Peri-renal Group - Control Peri-ovarian Group; Induced Peri-ovarian Group - Control Peri-renal Group; Control Peri-renal Group - Induced Peri-renal Group; Induced Peri-ovarian Group - Induced Peri-renal Group |
| L-Proline | 4.42E+31 | 3.26E-125 | 4.05E-124 | Control Peri-renal Group - Control Peri-ovarian Group; Induced Peri-ovarian Group - Control Peri-ovarian Group; Induced Peri-renal Group - Control Peri-ovarian Group; Induced Peri-ovarian Group - Control Peri-renal Group; Induced Peri-renal Group - Control Peri-renal Group; Induced Peri-ovarian Group - Induced Peri-renal Group |
| 2-Aminoheptanedioic acid | 3.08E+31 | 1.39E-124 | 1.51E-123 | Control Peri-renal Group - Control Peri-ovarian Group; Induced Peri-ovarian Group - Control Peri-ovarian Group; Induced Peri-renal Group - Control Peri-ovarian Group; Induced Peri-ovarian Group - Control Peri-renal Group; Control Peri-renal Group - Induced Peri-renal Group; Induced Peri-ovarian Group - Induced Peri-renal Group |
| Dopamine | 2.33E+31 | 4.24E-124 | 4.10E-123 | Control Peri-renal Group - Control Peri-ovarian Group; Induced Peri-ovarian Group - Control Peri-ovarian Group; Induced Peri-renal Group - Control Peri-ovarian Group; Induced Peri-ovarian Group - Control Peri-renal Group; Control Peri-renal Group - Induced Peri-renal Group; Induced Peri-ovarian Group - Induced Peri-renal Group |
| Citric acid | 2.06E+31 | 6.91E-124 | 6.01E-123 | Control Peri-renal Group - Control Peri-ovarian Group; Induced Peri-ovarian Group - Control Peri-ovarian Group; Induced Peri-renal Group - Control Peri-ovarian Group; Induced Peri-ovarian Group - Control Peri-renal Group; Control Peri-renal Group - Induced Peri-renal Group; Induced Peri-ovarian Group - Induced Peri-renal Group |
| Ethanolamine | 1.68E+31 | 1.55E-123 | 1.23E-122 | Control Peri-renal Group - Control Peri-ovarian Group; Induced Peri-ovarian Group - Control Peri-ovarian Group; Induced Peri-renal Group - Control Peri-ovarian Group; Induced Peri-ovarian Group - Control Peri-renal Group; Induced Peri-renal Group - Control Peri-renal Group; Induced Peri-ovarian Group - Induced Peri-renal Group |
| Uric acid | 1.60E+31 | 1.91E-123 | 1.38E-122 | Control Peri-renal Group - Control Peri-ovarian Group; Induced Peri-ovarian Group - Control Peri-ovarian Group; Induced Peri-renal Group - Control Peri-ovarian Group; Induced Peri-ovarian Group - Control Peri-renal Group; Induced Peri-renal Group - Control Peri-renal Group; Induced Peri-ovarian Group - Induced Peri-renal Group |
| D-Galactose | 1.24E+31 | 5.19E-123 | 3.47E-122 | Control Peri-renal Group - Control Peri-ovarian Group; Induced Peri-ovarian Group - Control Peri-ovarian Group; Induced Peri-renal Group - Control Peri-ovarian Group; Control Peri-renal Group - Induced Peri-ovarian Group; Induced Peri-renal Group - Control Peri-renal Group; Induced Peri-renal Group - Induced Peri-ovarian Group |
| Pectin | 7.09E+30 | 4.92E-122 | 2.86E-121 | Control Peri-ovarian Group - Control Peri-renal Group; Induced Peri-ovarian Group - Control Peri-ovarian Group; Induced Peri-renal Group - Control Peri-ovarian Group; Induced Peri-ovarian Group - Control Peri-renal Group; Induced Peri-renal Group - Control Peri-renal Group; Induced Peri-ovarian Group - Induced Peri-renal Group |
| L-Fucose | 7.09E+30 | 4.92E-122 | 2.86E-121 | Control Peri-ovarian Group - Control Peri-renal Group; Induced Peri-ovarian Group - Control Peri-ovarian Group; Induced Peri-renal Group - Control Peri-ovarian Group; Induced Peri-ovarian Group - Control Peri-renal Group; Induced Peri-renal Group - Control Peri-renal Group; Induced Peri-ovarian Group - Induced Peri-renal Group |
| Sorbitol | 5.72E+30 | 1.16E-121 | 6.33E-121 | Control Peri-renal Group - Control Peri-ovarian Group; Induced Peri-ovarian Group - Control Peri-ovarian Group; Induced Peri-renal Group - Control Peri-ovarian Group; Induced Peri-ovarian Group - Control Peri-renal Group; Induced Peri-renal Group - Control Peri-renal Group; Induced Peri-ovarian Group - Induced Peri-renal Group |
| Glycerol 1-octadecanoate | 5.24E+30 | 1.65E-121 | 8.46E-121 | Control Peri-renal Group - Control Peri-ovarian Group; Induced Peri-ovarian Group - Control Peri-ovarian Group; Induced Peri-renal Group - Control Peri-ovarian Group; Induced Peri-ovarian Group - Control Peri-renal Group; Control Peri-renal Group - Induced Peri-renal Group; Induced Peri-ovarian Group - Induced Peri-renal Group |
| L-Lactic acid | 4.94E+30 | 2.09E-121 | 1.01E-120 | Control Peri-renal Group - Control Peri-ovarian Group; Induced Peri-ovarian Group - Control Peri-ovarian Group; Induced Peri-renal Group - Control Peri-ovarian Group; Induced Peri-ovarian Group - Control Peri-renal Group; Induced Peri-renal Group - Control Peri-renal Group; Induced Peri-ovarian Group - Induced Peri-renal Group |
| Sucrose | 4.83E+30 | 2.29E-121 | 1.05E-120 | Control Peri-renal Group - Control Peri-ovarian Group; Induced Peri-ovarian Group - Control Peri-ovarian Group; Induced Peri-renal Group - Control Peri-ovarian Group; Induced Peri-ovarian Group - Control Peri-renal Group; Induced Peri-renal Group - Control Peri-renal Group; Induced Peri-renal Group - Induced Peri-ovarian Group |
| Hydroxyphenyllactic acid | 4.59E+30 | 2.80E-121 | 1.22E-120 | Control Peri-renal Group - Control Peri-ovarian Group; Induced Peri-ovarian Group - Control Peri-ovarian Group; Induced Peri-renal Group - Control Peri-ovarian Group; Induced Peri-ovarian Group - Control Peri-renal Group; Control Peri-renal Group - Induced Peri-renal Group; Induced Peri-ovarian Group - Induced Peri-renal Group |
| Galacturonic acid | 4.44E+30 | 3.22E-121 | 1.33E-120 | Control Peri-renal Group - Control Peri-ovarian Group; Induced Peri-ovarian Group - Control Peri-ovarian Group; Induced Peri-renal Group - Control Peri-ovarian Group; Induced Peri-ovarian Group - Control Peri-renal Group; Control Peri-renal Group - Induced Peri-renal Group; Induced Peri-ovarian Group - Induced Peri-renal Group |
| Glycine | 3.58E+30 | 7.61E-121 | 3.01E-120 | Control Peri-renal Group - Control Peri-ovarian Group; Induced Peri-ovarian Group - Control Peri-ovarian Group; Induced Peri-renal Group - Control Peri-ovarian Group; Induced Peri-ovarian Group - Control Peri-renal Group; Induced Peri-renal Group - Control Peri-renal Group; Induced Peri-ovarian Group - Induced Peri-renal Group |
| Allose | 3.38E+30 | 9.51E-121 | 3.31E-120 | Control Peri-ovarian Group - Control Peri-renal Group; Control Peri-ovarian Group - Induced Peri-ovarian Group; Control Peri-ovarian Group - Induced Peri-renal Group; Control Peri-renal Group - Induced Peri-ovarian Group; Control Peri-renal Group - Induced Peri-renal Group; Induced Peri-ovarian Group - Induced Peri-renal Group |
| Gluconolactone | 3.38E+30 | 9.51E-121 | 3.31E-120 | Control Peri-ovarian Group - Control Peri-renal Group; Control Peri-ovarian Group - Induced Peri-ovarian Group; Control Peri-ovarian Group - Induced Peri-renal Group; Control Peri-renal Group - Induced Peri-ovarian Group; Control Peri-renal Group - Induced Peri-renal Group; Induced Peri-ovarian Group - Induced Peri-renal Group |
| D-Mannose | 3.38E+30 | 9.51E-121 | 3.31E-120 | Control Peri-ovarian Group - Control Peri-renal Group; Control Peri-ovarian Group - Induced Peri-ovarian Group; Control Peri-ovarian Group - Induced Peri-renal Group; Control Peri-renal Group - Induced Peri-ovarian Group; Control Peri-renal Group - Induced Peri-renal Group; Induced Peri-ovarian Group - Induced Peri-renal Group |
| Taurine | 3.26E+30 | 1.10E-120 | 3.67E-120 | Control Peri-renal Group - Control Peri-ovarian Group; Induced Peri-ovarian Group - Control Peri-ovarian Group; Induced Peri-renal Group - Control Peri-ovarian Group; Induced Peri-ovarian Group - Control Peri-renal Group; Induced Peri-renal Group - Control Peri-renal Group; Induced Peri-ovarian Group - Induced Peri-renal Group |
| Fructose 1-phosphate | 3.15E+30 | 1.26E-120 | 4.02E-120 | Control Peri-renal Group - Control Peri-ovarian Group; Induced Peri-ovarian Group - Control Peri-ovarian Group; Induced Peri-renal Group - Control Peri-ovarian Group; Induced Peri-ovarian Group - Control Peri-renal Group; Control Peri-renal Group - Induced Peri-renal Group; Induced Peri-ovarian Group - Induced Peri-renal Group |
| Isobutyrylglycine | 3.13E+30 | 1.29E-120 | 4.02E-120 | Control Peri-ovarian Group - Control Peri-renal Group; Induced Peri-ovarian Group - Control Peri-ovarian Group; Induced Peri-renal Group - Control Peri-ovarian Group; Induced Peri-ovarian Group - Control Peri-renal Group; Induced Peri-renal Group - Control Peri-renal Group; Induced Peri-ovarian Group - Induced Peri-renal Group |
| Sarcosine | 2.40E+30 | 3.75E-120 | 1.13E-119 | Control Peri-renal Group - Control Peri-ovarian Group; Induced Peri-ovarian Group - Control Peri-ovarian Group; Induced Peri-renal Group - Control Peri-ovarian Group; Induced Peri-ovarian Group - Control Peri-renal Group; Induced Peri-renal Group - Control Peri-renal Group; Induced Peri-ovarian Group - Induced Peri-renal Group |
| L-Isoleucine | 2.11E+30 | 6.28E-120 | 1.71E-119 | Control Peri-renal Group - Control Peri-ovarian Group; Induced Peri-ovarian Group - Control Peri-ovarian Group; Induced Peri-renal Group - Control Peri-ovarian Group; Induced Peri-ovarian Group - Control Peri-renal Group; Induced Peri-renal Group - Control Peri-renal Group; Induced Peri-ovarian Group - Induced Peri-renal Group |
| L-Alloisoleucine | 2.11E+30 | 6.28E-120 | 1.71E-119 | Control Peri-renal Group - Control Peri-ovarian Group; Induced Peri-ovarian Group - Control Peri-ovarian Group; Induced Peri-renal Group - Control Peri-ovarian Group; Induced Peri-ovarian Group - Control Peri-renal Group; Induced Peri-renal Group - Control Peri-renal Group; Induced Peri-ovarian Group - Induced Peri-renal Group |
| Gamma-Aminobutyric acid | 2.11E+30 | 6.28E-120 | 1.71E-119 | Control Peri-renal Group - Control Peri-ovarian Group; Induced Peri-ovarian Group - Control Peri-ovarian Group; Induced Peri-renal Group - Control Peri-ovarian Group; Induced Peri-ovarian Group - Control Peri-renal Group; Induced Peri-renal Group - Control Peri-renal Group; Induced Peri-ovarian Group - Induced Peri-renal Group |
| D-Glucose | 1.48E+30 | 2.61E-119 | 6.88E-119 | Control Peri-renal Group - Control Peri-ovarian Group; Induced Peri-ovarian Group - Control Peri-ovarian Group; Induced Peri-renal Group - Control Peri-ovarian Group; Induced Peri-ovarian Group - Control Peri-renal Group; Induced Peri-renal Group - Control Peri-renal Group; Induced Peri-ovarian Group - Induced Peri-renal Group |
| L-Arginine | 1.43E+30 | 3.00E-119 | 7.68E-119 | Control Peri-renal Group - Control Peri-ovarian Group; Induced Peri-ovarian Group - Control Peri-ovarian Group; Control Peri-ovarian Group - Induced Peri-renal Group; Induced Peri-ovarian Group - Control Peri-renal Group; Control Peri-renal Group - Induced Peri-renal Group; Induced Peri-ovarian Group - Induced Peri-renal Group |
| Pyruvic acid | 1.41E+30 | 3.15E-119 | 7.83E-119 | Control Peri-renal Group - Control Peri-ovarian Group; Induced Peri-ovarian Group - Control Peri-ovarian Group; Induced Peri-renal Group - Control Peri-ovarian Group; Induced Peri-ovarian Group - Control Peri-renal Group; Induced Peri-renal Group - Control Peri-renal Group; Induced Peri-renal Group - Induced Peri-ovarian Group |
| O-Acetylserine | 1.40E+30 | 3.24E-119 | 7.83E-119 | Control Peri-renal Group - Control Peri-ovarian Group; Induced Peri-ovarian Group - Control Peri-ovarian Group; Induced Peri-renal Group - Control Peri-ovarian Group; Induced Peri-ovarian Group - Control Peri-renal Group; Induced Peri-renal Group - Control Peri-renal Group; Induced Peri-ovarian Group - Induced Peri-renal Group |
| Uracil | 1.26E+30 | 4.93E-119 | 1.16E-118 | Control Peri-ovarian Group - Control Peri-renal Group; Induced Peri-ovarian Group - Control Peri-ovarian Group; Control Peri-ovarian Group - Induced Peri-renal Group; Induced Peri-ovarian Group - Control Peri-renal Group; Control Peri-renal Group - Induced Peri-renal Group; Induced Peri-ovarian Group - Induced Peri-renal Group |
| 4-Hydroxyphenylpyruvic acid | 1.13E+30 | 7.67E-119 | 1.76E-118 | Control Peri-renal Group - Control Peri-ovarian Group; Induced Peri-ovarian Group - Control Peri-ovarian Group; Induced Peri-renal Group - Control Peri-ovarian Group; Induced Peri-ovarian Group - Control Peri-renal Group; Induced Peri-renal Group - Control Peri-renal Group; Induced Peri-ovarian Group - Induced Peri-renal Group |
| Pyroglutamic acid | 9.41E+29 | 1.59E-118 | 3.54E-118 | Control Peri-renal Group - Control Peri-ovarian Group; Induced Peri-ovarian Group - Control Peri-ovarian Group; Induced Peri-renal Group - Control Peri-ovarian Group; Induced Peri-ovarian Group - Control Peri-renal Group; Induced Peri-renal Group - Control Peri-renal Group; Induced Peri-ovarian Group - Induced Peri-renal Group |
| D-Galactosamine | 9.23E+29 | 1.72E-118 | 3.73E-118 | Control Peri-ovarian Group - Control Peri-renal Group; Induced Peri-ovarian Group - Control Peri-ovarian Group; Control Peri-ovarian Group - Induced Peri-renal Group; Induced Peri-ovarian Group - Control Peri-renal Group; Control Peri-renal Group - Induced Peri-renal Group; Induced Peri-ovarian Group - Induced Peri-renal Group |
| Urea | 7.28E+29 | 4.43E-118 | 9.26E-118 | Control Peri-renal Group - Control Peri-ovarian Group; Induced Peri-ovarian Group - Control Peri-ovarian Group; Induced Peri-renal Group - Control Peri-ovarian Group; Control Peri-renal Group - Induced Peri-ovarian Group; Induced Peri-renal Group - Control Peri-renal Group; Induced Peri-renal Group - Induced Peri-ovarian Group |
| L-Sorbose | 7.26E+29 | 4.47E-118 | 9.26E-118 | Control Peri-renal Group - Control Peri-ovarian Group; Induced Peri-ovarian Group - Control Peri-ovarian Group; Induced Peri-renal Group - Control Peri-ovarian Group; Induced Peri-ovarian Group - Control Peri-renal Group; Induced Peri-renal Group - Control Peri-renal Group; Induced Peri-ovarian Group - Induced Peri-renal Group |
| Glycerol 3-phosphate | 7.05E+29 | 5.04E-118 | 1.02E-117 | Control Peri-renal Group - Control Peri-ovarian Group; Induced Peri-ovarian Group - Control Peri-ovarian Group; Induced Peri-renal Group - Control Peri-ovarian Group; Induced Peri-ovarian Group - Control Peri-renal Group; Control Peri-renal Group - Induced Peri-renal Group; Induced Peri-ovarian Group - Induced Peri-renal Group |
| Alpha-Lactose | 6.58E+29 | 6.64E-118 | 1.31E-117 | Control Peri-renal Group - Control Peri-ovarian Group; Induced Peri-ovarian Group - Control Peri-ovarian Group; Induced Peri-renal Group - Control Peri-ovarian Group; Induced Peri-ovarian Group - Control Peri-renal Group; Induced Peri-renal Group - Control Peri-renal Group; Induced Peri-ovarian Group - Induced Peri-renal Group |
| Galactitol | 6.06E+29 | 9.20E-118 | 1.78E-117 | Control Peri-renal Group - Control Peri-ovarian Group; Control Peri-ovarian Group - Induced Peri-ovarian Group; Control Peri-ovarian Group - Induced Peri-renal Group; Control Peri-renal Group - Induced Peri-ovarian Group; Control Peri-renal Group - Induced Peri-renal Group; Induced Peri-renal Group - Induced Peri-ovarian Group |
| N-Acetylmannosamine | 5.13E+29 | 1.80E-117 | 3.40E-117 | Control Peri-renal Group - Control Peri-ovarian Group; Induced Peri-ovarian Group - Control Peri-ovarian Group; Induced Peri-renal Group - Control Peri-ovarian Group; Induced Peri-ovarian Group - Control Peri-renal Group; Induced Peri-renal Group - Control Peri-renal Group; Induced Peri-ovarian Group - Induced Peri-renal Group |
| Arabinose | 4.91E+29 | 2.15E-117 | 3.98E-117 | Control Peri-ovarian Group - Control Peri-renal Group; Induced Peri-ovarian Group - Control Peri-ovarian Group; Induced Peri-renal Group - Control Peri-ovarian Group; Induced Peri-ovarian Group - Control Peri-renal Group; Induced Peri-renal Group - Control Peri-renal Group; Induced Peri-ovarian Group - Induced Peri-renal Group |
| Propionylglycine | 4.73E+29 | 2.49E-117 | 4.51E-117 | Control Peri-renal Group - Control Peri-ovarian Group; Induced Peri-ovarian Group - Control Peri-ovarian Group; Induced Peri-renal Group - Control Peri-ovarian Group; Induced Peri-ovarian Group - Control Peri-renal Group; Induced Peri-renal Group - Control Peri-renal Group; Induced Peri-ovarian Group - Induced Peri-renal Group |
| Tryptamine | 4.19E+29 | 4.03E-117 | 6.99E-117 | Control Peri-renal Group - Control Peri-ovarian Group; Induced Peri-ovarian Group - Control Peri-ovarian Group; Control Peri-ovarian Group - Induced Peri-renal Group; Induced Peri-ovarian Group - Control Peri-renal Group; Control Peri-renal Group - Induced Peri-renal Group; Induced Peri-ovarian Group - Induced Peri-renal Group |
| (S)-b-aminoisobutyric acid | 4.17E+29 | 4.10E-117 | 6.99E-117 | Control Peri-renal Group - Control Peri-ovarian Group; Induced Peri-ovarian Group - Control Peri-ovarian Group; Induced Peri-renal Group - Control Peri-ovarian Group; Induced Peri-ovarian Group - Control Peri-renal Group; Induced Peri-renal Group - Control Peri-renal Group; Induced Peri-ovarian Group - Induced Peri-renal Group |
| L-Valine | 4.17E+29 | 4.10E-117 | 6.99E-117 | Control Peri-renal Group - Control Peri-ovarian Group; Induced Peri-ovarian Group - Control Peri-ovarian Group; Induced Peri-renal Group - Control Peri-ovarian Group; Induced Peri-ovarian Group - Control Peri-renal Group; Induced Peri-renal Group - Control Peri-renal Group; Induced Peri-ovarian Group - Induced Peri-renal Group |
| Oxalic acid | 3.92E+29 | 5.25E-117 | 8.46E-117 | Control Peri-renal Group - Control Peri-ovarian Group; Induced Peri-ovarian Group - Control Peri-ovarian Group; Induced Peri-renal Group - Control Peri-ovarian Group; Induced Peri-ovarian Group - Control Peri-renal Group; Induced Peri-renal Group - Control Peri-renal Group; Induced Peri-ovarian Group - Induced Peri-renal Group |
| 2-Hydroxybutyric acid | 3.92E+29 | 5.25E-117 | 8.46E-117 | Control Peri-renal Group - Control Peri-ovarian Group; Induced Peri-ovarian Group - Control Peri-ovarian Group; Induced Peri-renal Group - Control Peri-ovarian Group; Induced Peri-ovarian Group - Control Peri-renal Group; Induced Peri-renal Group - Control Peri-renal Group; Induced Peri-ovarian Group - Induced Peri-renal Group |
| Hydroxypropionic acid | 3.92E+29 | 5.25E-117 | 8.46E-117 | Control Peri-renal Group - Control Peri-ovarian Group; Induced Peri-ovarian Group - Control Peri-ovarian Group; Induced Peri-renal Group - Control Peri-ovarian Group; Induced Peri-ovarian Group - Control Peri-renal Group; Induced Peri-renal Group - Control Peri-renal Group; Induced Peri-ovarian Group - Induced Peri-renal Group |
| Ornithine | 3.58E+29 | 7.60E-117 | 1.20E-116 | Control Peri-renal Group - Control Peri-ovarian Group; Induced Peri-ovarian Group - Control Peri-ovarian Group; Induced Peri-renal Group - Control Peri-ovarian Group; Control Peri-renal Group - Induced Peri-ovarian Group; Induced Peri-renal Group - Control Peri-renal Group; Induced Peri-renal Group - Induced Peri-ovarian Group |
| Mannose 6-phosphate | 3.29E+29 | 1.06E-116 | 1.65E-116 | Control Peri-ovarian Group - Control Peri-renal Group; Control Peri-ovarian Group - Induced Peri-ovarian Group; Control Peri-ovarian Group - Induced Peri-renal Group; Induced Peri-ovarian Group - Control Peri-renal Group; Induced Peri-renal Group - Control Peri-renal Group; Induced Peri-ovarian Group - Induced Peri-renal Group |
| Heptadecanoic acid | 2.89E+29 | 1.78E-116 | 2.66E-116 | Control Peri-ovarian Group - Control Peri-renal Group; Control Peri-ovarian Group - Induced Peri-ovarian Group; Induced Peri-renal Group - Control Peri-ovarian Group; Control Peri-renal Group - Induced Peri-ovarian Group; Induced Peri-renal Group - Control Peri-renal Group; Induced Peri-renal Group - Induced Peri-ovarian Group |
| Octadecanol | 2.89E+29 | 1.78E-116 | 2.66E-116 | Control Peri-ovarian Group - Control Peri-renal Group; Control Peri-ovarian Group - Induced Peri-ovarian Group; Induced Peri-renal Group - Control Peri-ovarian Group; Control Peri-renal Group - Induced Peri-ovarian Group; Induced Peri-renal Group - Control Peri-renal Group; Induced Peri-renal Group - Induced Peri-ovarian Group |
| 3-Dehydroquinate | 2.51E+29 | 3.12E-116 | 4.60E-116 | Control Peri-renal Group - Control Peri-ovarian Group; Induced Peri-ovarian Group - Control Peri-ovarian Group; Induced Peri-renal Group - Control Peri-ovarian Group; Induced Peri-ovarian Group - Control Peri-renal Group; Control Peri-renal Group - Induced Peri-renal Group; Induced Peri-ovarian Group - Induced Peri-renal Group |
| D-Glucuronic acid | 2.35E+29 | 4.06E-116 | 5.89E-116 | Control Peri-ovarian Group - Control Peri-renal Group; Induced Peri-ovarian Group - Control Peri-ovarian Group; Induced Peri-renal Group - Control Peri-ovarian Group; Induced Peri-ovarian Group - Control Peri-renal Group; Induced Peri-renal Group - Control Peri-renal Group; Induced Peri-ovarian Group - Induced Peri-renal Group |
| Coniferyl aldehyde | 1.96E+29 | 8.36E-116 | 1.19E-115 | Control Peri-renal Group - Control Peri-ovarian Group; Induced Peri-ovarian Group - Control Peri-ovarian Group; Induced Peri-renal Group - Control Peri-ovarian Group; Induced Peri-ovarian Group - Control Peri-renal Group; Control Peri-renal Group - Induced Peri-renal Group; Induced Peri-ovarian Group - Induced Peri-renal Group |
| L-Octanoylcarnitine | 1.93E+29 | 8.93E-116 | 1.25E-115 | Control Peri-ovarian Group - Control Peri-renal Group; Induced Peri-ovarian Group - Control Peri-ovarian Group; Control Peri-ovarian Group - Induced Peri-renal Group; Induced Peri-ovarian Group - Control Peri-renal Group; Control Peri-renal Group - Induced Peri-renal Group; Induced Peri-ovarian Group - Induced Peri-renal Group |
| D-Maltose | 1.84E+29 | 1.09E-115 | 1.50E-115 | Control Peri-renal Group - Control Peri-ovarian Group; Induced Peri-ovarian Group - Control Peri-ovarian Group; Induced Peri-renal Group - Control Peri-ovarian Group; Induced Peri-ovarian Group - Control Peri-renal Group; Control Peri-renal Group - Induced Peri-renal Group; Induced Peri-ovarian Group - Induced Peri-renal Group |
| L-Malic acid | 1.75E+29 | 1.33E-115 | 1.80E-115 | Control Peri-renal Group - Control Peri-ovarian Group; Induced Peri-ovarian Group - Control Peri-ovarian Group; Induced Peri-renal Group - Control Peri-ovarian Group; Induced Peri-ovarian Group - Control Peri-renal Group; Induced Peri-renal Group - Control Peri-renal Group; Induced Peri-ovarian Group - Induced Peri-renal Group |
| Dihydroxyacetone phosphate | 1.75E+29 | 1.34E-115 | 1.80E-115 | Control Peri-renal Group - Control Peri-ovarian Group; Induced Peri-ovarian Group - Control Peri-ovarian Group; Induced Peri-renal Group - Control Peri-ovarian Group; Induced Peri-ovarian Group - Control Peri-renal Group; Induced Peri-renal Group - Control Peri-renal Group; Induced Peri-ovarian Group - Induced Peri-renal Group |
| D-Tagatose | 1.74E+29 | 1.36E-115 | 1.80E-115 | Control Peri-renal Group - Control Peri-ovarian Group; Induced Peri-ovarian Group - Control Peri-ovarian Group; Induced Peri-renal Group - Control Peri-ovarian Group; Induced Peri-ovarian Group - Control Peri-renal Group; Induced Peri-renal Group - Control Peri-renal Group; Induced Peri-ovarian Group - Induced Peri-renal Group |
| L-Threonine | 1.46E+29 | 2.75E-115 | 3.58E-115 | Control Peri-renal Group - Control Peri-ovarian Group; Induced Peri-ovarian Group - Control Peri-ovarian Group; Induced Peri-renal Group - Control Peri-ovarian Group; Induced Peri-ovarian Group - Control Peri-renal Group; Induced Peri-renal Group - Control Peri-renal Group; Induced Peri-ovarian Group - Induced Peri-renal Group |
| L-Phenylalanine | 1.25E+29 | 5.08E-115 | 6.49E-115 | Control Peri-renal Group - Control Peri-ovarian Group; Induced Peri-ovarian Group - Control Peri-ovarian Group; Induced Peri-renal Group - Control Peri-ovarian Group; Induced Peri-ovarian Group - Control Peri-renal Group; Induced Peri-renal Group - Control Peri-renal Group; Induced Peri-ovarian Group - Induced Peri-renal Group |
| N-Acetylornithine | 1.02E+29 | 1.17E-114 | 1.48E-114 | Control Peri-renal Group - Control Peri-ovarian Group; Induced Peri-ovarian Group - Control Peri-ovarian Group; Control Peri-ovarian Group - Induced Peri-renal Group; Induced Peri-ovarian Group - Control Peri-renal Group; Control Peri-renal Group - Induced Peri-renal Group; Induced Peri-ovarian Group - Induced Peri-renal Group |
| Benzoic acid | 7.62E+28 | 3.69E-114 | 4.59E-114 | Control Peri-ovarian Group - Control Peri-renal Group; Induced Peri-ovarian Group - Control Peri-ovarian Group; Control Peri-ovarian Group - Induced Peri-renal Group; Induced Peri-ovarian Group - Control Peri-renal Group; Induced Peri-renal Group - Control Peri-renal Group; Induced Peri-ovarian Group - Induced Peri-renal Group |
| L-Cysteine | 5.99E+28 | 9.64E-114 | 1.18E-113 | Control Peri-renal Group - Control Peri-ovarian Group; Induced Peri-ovarian Group - Control Peri-ovarian Group; Induced Peri-renal Group - Control Peri-ovarian Group; Control Peri-renal Group - Induced Peri-ovarian Group; Control Peri-renal Group - Induced Peri-renal Group; Induced Peri-renal Group - Induced Peri-ovarian Group |
| Myoinositol | 5.94E+28 | 1.00E-113 | 1.21E-113 | Control Peri-renal Group - Control Peri-ovarian Group; Induced Peri-ovarian Group - Control Peri-ovarian Group; Induced Peri-renal Group - Control Peri-ovarian Group; Induced Peri-ovarian Group - Control Peri-renal Group; Induced Peri-renal Group - Control Peri-renal Group; Induced Peri-ovarian Group - Induced Peri-renal Group |
| Indoleacetic acid | 4.99E+28 | 2.01E-113 | 2.39E-113 | Control Peri-renal Group - Control Peri-ovarian Group; Induced Peri-ovarian Group - Control Peri-ovarian Group; Control Peri-ovarian Group - Induced Peri-renal Group; Induced Peri-ovarian Group - Control Peri-renal Group; Control Peri-renal Group - Induced Peri-renal Group; Induced Peri-ovarian Group - Induced Peri-renal Group |
| Palmitic acid | 4.94E+28 | 2.09E-113 | 2.46E-113 | Control Peri-ovarian Group - Control Peri-renal Group; Induced Peri-ovarian Group - Control Peri-ovarian Group; Induced Peri-renal Group - Control Peri-ovarian Group; Induced Peri-ovarian Group - Control Peri-renal Group; Induced Peri-renal Group - Control Peri-renal Group; Induced Peri-renal Group - Induced Peri-ovarian Group |
| Mannitol | 4.39E+28 | 3.36E-113 | 3.90E-113 | Control Peri-renal Group - Control Peri-ovarian Group; Induced Peri-ovarian Group - Control Peri-ovarian Group; Induced Peri-renal Group - Control Peri-ovarian Group; Induced Peri-ovarian Group - Control Peri-renal Group; Induced Peri-renal Group - Control Peri-renal Group; Induced Peri-ovarian Group - Induced Peri-renal Group |
| N-Acetylglutamine | 3.85E+28 | 5.65E-113 | 6.46E-113 | Control Peri-renal Group - Control Peri-ovarian Group; Induced Peri-ovarian Group - Control Peri-ovarian Group; Induced Peri-renal Group - Control Peri-ovarian Group; Induced Peri-ovarian Group - Control Peri-renal Group; Induced Peri-renal Group - Control Peri-renal Group; Induced Peri-ovarian Group - Induced Peri-renal Group |
| Ascorbic acid | 3.17E+28 | 1.23E-112 | 1.39E-112 | Control Peri-renal Group - Control Peri-ovarian Group; Induced Peri-ovarian Group - Control Peri-ovarian Group; Induced Peri-renal Group - Control Peri-ovarian Group; Induced Peri-ovarian Group - Control Peri-renal Group; Control Peri-renal Group - Induced Peri-renal Group; Induced Peri-ovarian Group - Induced Peri-renal Group |
| Coniferyl alcohol | 2.69E+28 | 2.38E-112 | 2.66E-112 | Control Peri-renal Group - Control Peri-ovarian Group; Induced Peri-ovarian Group - Control Peri-ovarian Group; Induced Peri-renal Group - Control Peri-ovarian Group; Induced Peri-ovarian Group - Control Peri-renal Group; Control Peri-renal Group - Induced Peri-renal Group; Induced Peri-ovarian Group - Induced Peri-renal Group |
| Ureidosuccinic acid | 1.99E+28 | 7.94E-112 | 8.74E-112 | Control Peri-renal Group - Control Peri-ovarian Group; Induced Peri-ovarian Group - Control Peri-ovarian Group; Induced Peri-renal Group - Control Peri-ovarian Group; Induced Peri-ovarian Group - Control Peri-renal Group; Control Peri-renal Group - Induced Peri-renal Group; Induced Peri-ovarian Group - Induced Peri-renal Group |
| Succinic acid | 1.76E+28 | 1.30E-111 | 1.41E-111 | Control Peri-ovarian Group - Control Peri-renal Group; Induced Peri-ovarian Group - Control Peri-ovarian Group; Control Peri-ovarian Group - Induced Peri-renal Group; Induced Peri-ovarian Group - Control Peri-renal Group; Control Peri-renal Group - Induced Peri-renal Group; Induced Peri-ovarian Group - Induced Peri-renal Group |
| Glucosamine | 1.14E+28 | 7.49E-111 | 8.05E-111 | Control Peri-renal Group - Control Peri-ovarian Group; Induced Peri-ovarian Group - Control Peri-ovarian Group; Induced Peri-renal Group - Control Peri-ovarian Group; Induced Peri-ovarian Group - Control Peri-renal Group; Induced Peri-renal Group - Control Peri-renal Group; Induced Peri-ovarian Group - Induced Peri-renal Group |
| D-Glucurono-6,3-lactone | 8.28E+27 | 2.65E-110 | 2.81E-110 | Control Peri-renal Group - Control Peri-ovarian Group; Induced Peri-ovarian Group - Control Peri-ovarian Group; Induced Peri-renal Group - Control Peri-ovarian Group; Induced Peri-ovarian Group - Control Peri-renal Group; Induced Peri-renal Group - Control Peri-renal Group; Induced Peri-ovarian Group - Induced Peri-renal Group |
| Dodecanoic acid | 7.49E+27 | 3.95E-110 | 4.14E-110 | Control Peri-renal Group - Control Peri-ovarian Group; Control Peri-ovarian Group - Induced Peri-ovarian Group; Induced Peri-renal Group - Control Peri-ovarian Group; Control Peri-renal Group - Induced Peri-ovarian Group; Induced Peri-renal Group - Control Peri-renal Group; Induced Peri-renal Group - Induced Peri-ovarian Group |
| Stearic acid | 7.21E+27 | 4.60E-110 | 4.76E-110 | Control Peri-ovarian Group - Control Peri-renal Group; Induced Peri-ovarian Group - Control Peri-ovarian Group; Induced Peri-renal Group - Control Peri-ovarian Group; Induced Peri-ovarian Group - Control Peri-renal Group; Induced Peri-renal Group - Control Peri-renal Group; Induced Peri-renal Group - Induced Peri-ovarian Group |
| Phosphoric acid | 1.55E+27 | 2.16E-107 | 2.21E-107 | Control Peri-renal Group - Control Peri-ovarian Group; Induced Peri-ovarian Group - Control Peri-ovarian Group; Induced Peri-renal Group - Control Peri-ovarian Group; Induced Peri-ovarian Group - Control Peri-renal Group; Control Peri-renal Group - Induced Peri-renal Group; Induced Peri-ovarian Group - Induced Peri-renal Group |
| Pimelic acid | 1.37E+27 | 3.58E-107 | 3.62E-107 | Control Peri-ovarian Group - Control Peri-renal Group; Control Peri-ovarian Group - Induced Peri-ovarian Group; Induced Peri-renal Group - Control Peri-ovarian Group; Induced Peri-ovarian Group - Control Peri-renal Group; Induced Peri-renal Group - Control Peri-renal Group; Induced Peri-renal Group - Induced Peri-ovarian Group |
| Myristic acid | 8.40E+26 | 2.49E-106 | 2.49E-106 | Control Peri-renal Group - Control Peri-ovarian Group; Induced Peri-ovarian Group - Control Peri-ovarian Group; Induced Peri-renal Group - Control Peri-ovarian Group; Induced Peri-ovarian Group - Control Peri-renal Group; Induced Peri-renal Group - Control Peri-renal Group; Induced Peri-ovarian Group - Induced Peri-renal Group |
